# Supplementary material for: Fieldwork-based determination of design priorities for point-of-use drinking water quality sensors for use in resource-limited environments
Source: PLoS One. 2020 Jan 24;15(1):e0228140. doi: 10.1371/journal.pone.0228140 (PMC6980542; doi:10.1371/journal.pone.0228140)
Supplement: S1 Appendix — Comprises the following sections, containing thirteen tables and two figures: Locations of study sitesStakeholder meetings descriptionDemographic informationConjoint analysis study design and sample size calculationsConjoint analysis cost level selectionPotential use cases for improved point-of-use testingTabulated results for design workshops and conjoint analysis interviewsConjoint analysis with demographic interaction effects. (PDF) [file pone.0228140.s001.pdf]

S1 Appendix: Fieldwork-based Determination of Design Priorities for Point-of-Use Drinking Water Quality Sensors for Use in Resource-Limited Environments

Michael S. Bono Jr., Sydney Beasley, Emily Hanhauser,
A. John Hart, Rohit Karnik, Chintan Vaishnav

Contents

1 Locations of study sites **3**

2 Stakeholder meetings description **4**

3 Demographic information **5**

4 Conjoint analysis study design and sample size calculations **7**

5 Conjoint analysis cost level selection **10**

6 Potential use cases for improved point-of-use testing **11**

7 Tabulated results **13**
- 7.1 Design workshops 13
- 7.2 Conjoint analysis interviews 14

8 Conjoint analysis with demographic interaction effects **17**

List of Tables

A Locations of study sites used for fieldwork-based research in this study . . . 3
B Summary statistics for group design workshops . . . 5
C Summary statistics for the pilot conjoint analysis interviews . 5
D Summary statistics for the main population of the full conjoint analysis interviews . . . 6
E Study design for full conjoint analysis interviews. . . . 7
F Sample size calculations for the full conjoint analysis interviews 10
G Design priorities for each design workshop, as well as overall determined design priorities . . . 13
H Pilot conjoint analysis interview results . . . 14
I Full conjoint analysis interview results for the main population 15
J Full conjoint analysis interview results for the local contacts . 16
K Full conjoint analysis interview results for the entire population 17
L Parameter estimates for significant parameters from the consideration of demographic interactions for the full conjoint analysis interviews . . . 21
M Parameter estimates for all parameters from the consideration of demographic interactions for the full conjoint analysis interviews . . . 22

List of Figures

A Potential use cases identified for improved point-of-use water tests . . . 12
B Parameter estimates for reusability and corresponding significant demographic interaction effects for the full conjoint analysis interviews . . . 20

1 Locations of study sites

Table A. Locations of study sites used for fieldwork-based research in this study: stakeholder meetings; individual qualitative knowledge, attitude, practice interviews (denoted as KAP Interviews); group design workshops with rural end users (denoted as End User Design Workshops); group design workshops with NGO staff (denoted as NGO Design Workshops); pilot conjoint analysis interviews (denoted as Conjoint Analysis Pilot); and full conjoint analysis interviews (denoted as Conjoint Analysis Interviews). Note: An additional design workshop with end users at Ichatu (Ramgarh District, Jharkhand, 23.5 N, 85.6 E) was excluded from our results due to deviations from the planned protocol, and only a portion of the participants in the Shedashi and Ronhe design workshops finished the entire workshop procedure.

State	Village/City	District	Coordinates	Methods Used
Maharashtra	Pune	Pune	18.5 N, 73.8 E	Stakeholder meeting
	Kalamb	Pune	19.0 N, 74.0 E	Stakeholder meeting
	Shedashi	Raigad	18.7 N, 73.2 E	Stakeholder meetings, KAP Interviews, End User Design Workshop
	Vavoshi	Raigad	18.8 N, 73.2 E	Stakeholder meeting
	Narangi	Raigad	18.8 N, 73.2 E	Stakeholder meeting, NGO Design Workshop
	Mumbai	Mumbai City	19.0 N, 72.9 E	Stakeholder meetings
Jharkhand	Ranchi	Ranchi	23.4 N, 85.3 E	Stakeholder meetings
	Karanjtoli	Ranchi	23.3 N,85.0 E	Stakeholder meeting, End User Design Workshop
	Torpa	Khunti	23.1 N, 85.3 E	Stakeholder meetings, KAP Interviews, NGO Design Workshop
	Ronhe	Khunti	23.3 N, 85.2 E	End User Design Workshop, Conjoint Analysis Interviews
	Gopla	Khunti	22.9 N, 85.1 E	Conjoint Analysis Interviews
	Belkhara	Koderma	24.4 N, 85.5 E	Conjoint Analysis Interviews
	Jolhakarma	Koderma	24.4 N, 85.5 E	Conjoint Analysis Interviews
Uttarakhand	Dehradun	Dehradun	30.3 N, 78.0 E	Stakeholder meetings, Conjoint Analysis Pilot
	Hadam	Tehri Garhwal	30.4 N, 78.4 E	Conjoint Analysis Pilot
	Jadipani	Tehri Garhwal	30.4 N, 78.4 E	Conjoint Analysis Pilot, Conjoint Analysis Interviews
	Kuriyal Gaon	Tehri Garhwal	30.4 N, 78.4 E	Conjoint Analysis Interviews
	Chureddhar	Tehri Garhwal	30.4 N, 78.4 E	Stakeholder meeting, Conjoint Analysis Interviews

2 Stakeholder meetings description

In Maharashtra, we met with staff from the two governmental agencies responsible for water quality monitoring: the Groundwater Surveys Development Agency (GSDA), which monitors the quantity and quality of groundwater used for drinking, and the Pollution Control Board (PCB), which is primarily charged with ensuring industrial compliance regarding emission of anthropogenic air and water contaminants. We also met with local governmental officials by visiting a rural public health center (PHC) in Shedashi and meeting with members of the Gram Panchayat (village council) in Shedashi, Vavoshi, and Kalamb. In addition, we met with staff from an NGO (Rural Communes Center for Experiential Learning in Narangi, abbreviated as RC-CEL), that works with rural communities to provide training in agriculture, livelihoods, and water treatment, as well as staff from two nationwide NGOs working on water quality management and sanitation (WaterAid and Arghyam).

In Jharkhand, we met with staff from United Nations International Children's Emergency Fund (UNICEF), which is mandated with carrying out part of the state water quality monitoring, and staff from PRADAN, an NGO which works with communities to increase awareness regarding water quality management, agriculture, livelihoods, and community mobilization; and Action for Community Empowerment (ACE), an NGO which works with communities on sanitation projects. In addition, we met with several local government officials for the village of Karanjtoli, including the head of the village council (Gram Panchayat), head of the block (the administrative unit between the village and district level), and the local village official in charge of water quality monitoring, which in Jharkhand is referred to as the Jalsahiya. Finally, we met with a researcher at a local university (BIT-Mesra) to learn more about available techniques for detection of chemical and biological contamination in drinking water.

In Uttarakhand, we met with staff from Himmotthan, an NGO which has a Memorandum of Understanding (MOU) with the state government to provide training and oversee projects related to water, sanitation, agriculture, livelihoods, and community mobilization. In addition, we met with staff from the Himalayan Institute Hospital Trust (HIHT), which works with rural residents on healthcare and public health needs.

3 Demographic information

Table B. Summary statistics for group design workshops, both overall and for workshops where attribute preference was evaluated at the individual level and at the group level.

Statistic	Total		Individual Preference		Group Preference	
	N	%	N	%	N	%
**Total participants**	71	100	50	70	21	30
**Female Participants**	41	58	20	28	21	30
**Male Participants**	30	42	30	42	0	0
**End Users**	55	77	42	59	13	18
**NGO Staff**	16	23	8	11	8	11
**Maharashtra Residents**	13	18	13	18	0	0
**Jharkhand Residents**	58	82	37	52	21	30

Table C. Summary statistics for the pilot conjoint analysis interviews conducted in Uttarakhand.

Statistic	N	%
**Total Participants**	10	100
**Female Participants**	5	50
**Male Participants**	5	50
**End User Participants**	6	60
**Local Community Leaders**	1	10
**NGO Staff Participants**	4	40

Table D. Summary statistics for the main population of the full conjoint analysis interviews conducted in Uttarakhand and Jharkhand.

Category	Statistic	N	%
**Overall**	**Total Participants**	45	100
**Gender**	**Female**	33	73
	**Male**	12	27
**State**	**Uttarakhand**	25	56
	**Jharkhand**	21	47
**Community Role**	**Women's SHG Members**	29	64
	**Local Community Leaders**	6	13
	**Local NGO Staff**	8	18
**Education**	**None**	7	16
	**At least 10th standard**	24	53
**Water Source**	**Springs**	12	27
	**Village pumped water supply**	19	42
**Water Decision Method**	**Sensory**	26	58
	**Routine treatment**	6	13
	**Assume safe**	6	13
	**Aware of testing**	3	7
**Recruitment**	**Recruited via GMB**	28	62

4 Conjoint analysis study design and sample size calculations

Table E. Study design for full conjoint analysis interviews.

Survey	Choice Set	Option	Reusability	Output	Time to Results	Ingredient Addition	Cost, ₹
1	1	1	Reusable	Amount + Recommendation	Next Day	Add Liquid	50
1	1	2	Reusable	Amount + Recommendation	Same Day	Combined	100
1	2	1	Reusable	Amount + Recommendation	Same Day	Add Liquid	100
1	2	2	Disposable	Amount + Recommendation	Next Day	Combined	100
1	3	1	Reusable	Amount	Next Day	Add Liquid	50
1	3	2	Disposable	Amount	Same Day	Add Liquid	100
1	4	1	Disposable	Amount + Recommendation	Next Day	Combined	100
1	4	2	Disposable	Amount	Next Day	Add Liquid	50
1	5	1	Reusable	Amount + Recommendation	Next Day	Add Liquid	100
1	5	2	Disposable	Amount + Recommendation	Next Day	Combined	50
1	6	1	Disposable	Amount + Recommendation	Same Day	Combined	50
1	6	2	Disposable	Amount	Next Day	Combined	100
1	7	1	Disposable	Amount + Recommendation	Same Day	Add Liquid	50
1	7	2	Reusable	Amount	Same Day	Add Liquid	100
1	8	1	Disposable	Amount	Same Day	Add Liquid	50
1	8	2	Disposable	Amount	Next Day	Combined	100
2	9	1	Disposable	Amount + Recommendation	Same Day	Add Liquid	100
2	9	2	Reusable	Amount	Same Day	Combined	100
2	10	1	Disposable	Amount	Next Day	Add Liquid	50
2	10	2	Reusable	Amount + Recommendation	Next Day	Add Liquid	100

Table E – continued from previous page

Survey	Choice Set	Option	Reusability	Output	Time to Results	Ingredient Addition	Cost, ₹
2	11	1	Reusable	Amount + Recommendation	Next Day	Add Liquid	50
2	11	2	Disposable	Amount + Recommendation	Same Day	Combined	50
2	12	1	Reusable	Amount	Same Day	Combined	50
2	12	2	Reusable	Amount + Recommendation	Next Day	Add Liquid	50
2	13	1	Reusable	Amount	Same Day	Combined	100
2	13	2	Disposable	Amount + Recommendation	Same Day	Add Liquid	100
2	14	1	Disposable	Amount + Recommendation	Next Day	Combined	50
2	14	2	Reusable	Amount + Recommendation	Same Day	Combined	100
2	15	1	Disposable	Amount + Recommendation	Next Day	Add Liquid	100
2	15	2	Reusable	Amount + Recommendation	Same Day	Combined	100
2	16	1	Reusable	Amount	Next Day	Combined	50
2	16	2	Reusable	Amount + Recommendation	Next Day	Add Liquid	100
3	17	1	Disposable	Amount	Same Day	Add Liquid	100
3	17	2	Disposable	Amount + Recommendation	Next Day	Combined	100
3	18	1	Reusable	Amount	Same Day	Add Liquid	50
3	18	2	Reusable	Amount + Recommendation	Next Day	Combined	50
3	19	1	Reusable	Amount	Next Day	Add Liquid	100
3	19	2	Disposable	Amount + Recommendation	Same Day	Add Liquid	100
3	20	1	Reusable	Amount	Same Day	Add Liquid	100
3	20	2	Disposable	Amount	Same Day	Combined	50
3	21	1	Reusable	Amount	Next Day	Add Liquid	100
3	21	2	Disposable	Amount + Recommendation	Next Day	Combined	100
3	22	1	Disposable	Amount	Same Day	Combined	50
3	22	2	Reusable	Amount + Recommendation	Next Day	Combined	50

Table E – continued from previous page

Survey	Choice Set	Option	Reusability	Output	Time to Results	Ingredient Addition	Cost, ₹
3	23	1	Disposable	Amount	Same Day	Add Liquid	50
3	23	2	Reusable	Amount + Recommendation	Next Day	Add Liquid	50
3	24	1	Disposable	Amount + Recommendation	Next Day	Add Liquid	100
3	24	2	Reusable	Amount	Next Day	Add Liquid	50
4	25	1	Disposable	Amount	Next Day	Combined	50
4	25	2	Reusable	Amount	Same Day	Add Liquid	50
4	26	1	Disposable	Amount + Recommendation	Next Day	Combined	100
4	26	2	Reusable	Amount + Recommendation	Same Day	Combined	50
4	27	1	Disposable	Amount + Recommendation	Same Day	Combined	100
4	27	2	Reusable	Amount	Same Day	Combined	50
4	28	1	Reusable	Amount	Next Day	Combined	100
4	28	2	Reusable	Amount + Recommendation	Same Day	Combined	50
4	29	1	Disposable	Amount + Recommendation	Same Day	Combined	100
4	29	2	Disposable	Amount	Next Day	Combined	50
4	30	1	Reusable	Amount	Next Day	Add Liquid	50
4	30	2	Reusable	Amount	Same Day	Combined	100
4	31	1	Disposable	Amount	Same Day	Add Liquid	100
4	31	2	Disposable	Amount + Recommendation	Same Day	Combined	50
4	32	1	Disposable	Amount	Same Day	Combined	100
4	32	2	Reusable	Amount	Same Day	Add Liquid	50

Table F. Sample size calculations for the full conjoint analysis interviews, using the parameter estimates and standard errors from the pilot conjoint analysis interviews (N = 10) and the analysis as described in de Bekker-Grob et al.[1], yielding required sample sizes for two-tailed $\alpha = 0.05$ and statistical powers of 0.8 ($\beta = 0.2$) and 0.9 ($\beta = 0.1$).

Parameter	Estimate	Std. Error	Rel. Std. Dev.	N ($\beta = 0.2$)	N ($\beta = 0.1$)
**Reusability: Disposable**	-0.277	0.147	1.676	22	30
**Cost: High**	-0.327	0.164	1.591	20	27
**Output: Amount**	-0.273	0.163	1.890	28	38
**Time to results: Next day**	-0.263	0.162	1.953	30	40
**Ingredients: Add liquid**	-0.436	0.255	1.850	27	36
**Ingredients: Add tablet**	0.173	0.230	4.216	140	187

5 Conjoint analysis cost level selection

In selecting sensor cost levels (presented in Indian Rupees, ₹) it was necessary to specify cost levels for both the disposable test, where the entire cost is paid for each new test, and for the mostly reusable test, containing both a fixed and variable cost corresponding to the reusable and disposable components. For the pilot interviews, the lower cost levels were selected based on typical prices for existing H2S tests (₹50) and blood glucose meters (₹500 for reusable electronics, ₹10 for disposable strips) in the Indian market. The higher cost levels were selected based on typical prices for a disposable test capable of detecting bacteria at the WHO limit of detection of one culture-forming unit (cfu) per 100 mL (e.g. around ₹100 for the ChekNsee test, Rakiro Biotech, Navi Mumbai, Maharashtra, India) and a suggested maximum price of ₹1000 from an interview with local government officials in Karanjtoli, Jharkhand. For the full interviews, these costs were adjusted in order to make the reusability and cost attributes as orthogonal as possible in order to reduce any interference between them. We did this by ensuring that the cost per test was equivalent (either ₹50 or ₹100) for the disposable and mostly reusable tests after 20 tests, selected based on hypothetical use cases of testing water quarterly for five years or bi-annually for a decade.

6 Potential use cases for improved point-of-use testing

Government-mandated water quality testing in India is initiated at the village level, with implementation varying by state. In Maharashtra, male village-level officials referred to as Jalsurakshaks collect water samples for testing at local (block-level) laboratories, whereas in Jharkhand the corresponding officials are female, known as Jalsahiyas, and use field test kits for measurement of basic chemical parameters (pH, iron, fluoride, and nitrate). We learned from meeting with NGO staff (Arghyam, WaterAid) that existing field testing kits were used by NGOs before adoption into government standards, with adoption into government standards spurred by exposure to the test kits' validity and utility in NGO use. As we observed a widespread need for improved point-of-use testing among NGOs, we decided that for our initial primary use case we would focus on NGO-led behavior change interventions in order to enable the incorporation of point-of-use testing into evidence-based behavior change communication. Moreover, we identified potential use cases for other NGO activities throughout their water quality project life cycle, as well as use cases among the rural residents and other local stakeholders that engage with these NGOs (Fig A).

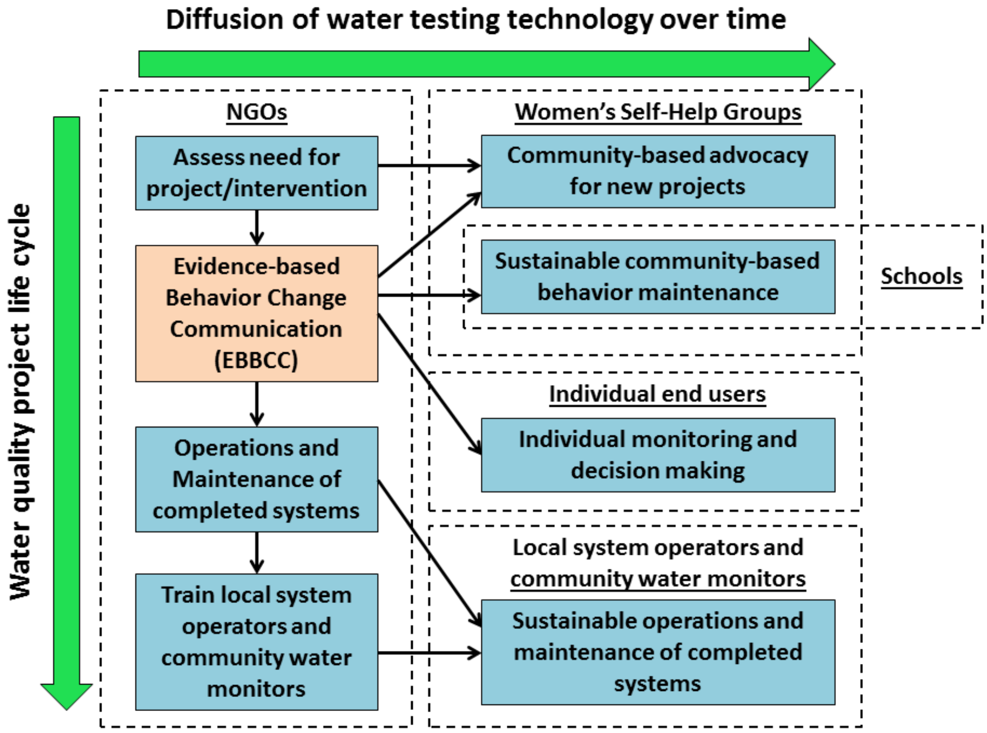

Fig A. Potential use cases identified for improved point-of-use bacterial water tests, beginning with initial use cases among NGOs working with resource-limited communities on water quality management with diffusion of technology to rural end users. Previously submitted as part of the unpublished provisional patent application Bono et al.[2]

7 Tabulated results

7.1 Design workshops

Table G. Design priorities for each design workshop, as well as overall determined design priorities calculated by assigning 5 points to the attribute with the highest priority, 4 points to the attribute with the second-highest priority, and 3 points to the attribute with the third-highest priority from each workshop.

Date (all 2016)	14-Jan	15-Jan	22-Jan	24-Jan	25-Jan	Overall score	Overall priority
Location	Narangi	Shedashi	Ronhe	Karanjtoli	Torpa	(#1=5pts,	
Participants	RC-CEL	End users	End users	End users	PRADAN	#2=4pts,	
Priority resolution	Individual	Individual	Group	Individual	Group	#3=3pts)	
Owned at community level					2	4	5 (tie)
Owned at household level	2 (tie)	1	1	1		19	1
Tells presence/absence			2			4	5 (tie)
Tells amount of contaminant							
Tells amount of contaminant and recommended action	1 (tie)	3		2 (tie)	1	17	2
Same-day/ immediate results	1 (tie)	2	3		3	15	3
Disposable							
Reusable	2 (tie)			3		7	4
No mixing required				2 (tie)		4	5 (tie)

7.2 Conjoint analysis interviews

Table H. Parameter estimates for pilot conjoint analysis interviews (N = 10), along with the standard error of the estimate, 95% confidence interval, p-value according to a likelihood ratio test, and significance level, where * denotes $p < 0.05$, for each level of each design attribute.

Attribute	Level	Estimate	Std. Error	95% CI	p-value	Significance
Reusability	Disposable	-0.28	0.15	[-0.57, 0.00]	0.039	*
	Mostly Reusable	0.28	0.15	[0.00, 0.57]		
Output	Amount	-0.27	0.16	[-0.60, 0.03]	0.063	
	Amount + Recommendation	0.27	0.16	[-0.03, 0.60]		
Time to results	Next Day	-0.26	0.16	[-0.58, 0.04]	0.077	
	Same Day	0.26	0.16	[-0.04, 0.58]		
Ingredients	Add Liquid	-0.44	0.26	[-0.95, 0.03]	0.156	
	Add Tablet	0.17	0.23	[-0.26, 0.63]		
	Combined	0.26	0.23	[-0.17, 0.72]		
Cost	High	-0.33	0.16	[-0.66, -0.02]	0.027	*
	Low	0.33	0.16	[0.02, 0.66]		

Table I. Parameter estimates for full conjoint analysis interviews of main population (N = 45, no local contacts), along with the standard error of the estimate, 95% confidence interval, p-value according to a likelihood ratio test, and significance level, where * denotes $p < 0.05$ and ** denotes $p < 0.005$, for each level of each design attribute.

Attribute	Level	Estimate	Std. Error	95% CI	p-value	Significance
Reusability	Disposable	-0.30	0.08	[-0.46, -0.14]	0.0001	**
	Mostly Reusable	0.30	0.08	[0.14, 0.46]		
Output	Amount	-0.68	0.09	[-0.86, -0.51]	<0.0001	**
	Amount + Recommendation	0.68	0.09	[0.51, 0.86]		
Time to results	Next Day	-0.27	0.08	[-0.44, -0.12]	0.0005	**
	Same Day	0.27	0.08	[0.12, 0.44]		
Ingredients	Add Liquid	-0.21	0.08	[-0.36, -0.06]	0.0060	*
	Combined	0.21	0.08	[0.06, 0.36]		
Cost per test, ₹	50	0.28	0.08	[0.12, 0.45]	0.0003	**
	100	-0.28	0.08	[-0.45, -0.12]		

Table J. Parameter estimates for full conjoint analysis interviews of local contacts (N = 8), along with the standard error of the estimate, 95% confidence interval, p-value according to a likelihood ratio test, and significance level, where * denotes $p < 0.05$, for each level of each design attribute.

Attribute	Level	Estimate	Std. Error	95% CI	p-value	Significance
Reusability	Disposable	0.19	0.18	[-0.15, 0.54]	0.266	
	Mostly Reusable	-0.19	0.18	[-0.54, 0.15]		
Output	Amount	-0.35	0.20	[-0.75, 0.02]	0.053	
	Amount + Recommendation	0.35	0.20	[-0.02, 0.75]		
Time to Results	Next Day	-0.40	0.19	[-0.78, -0.06]	0.017	*
	Same Day	0.40	0.19	[0.06, 0.78]		
Ingredients	Add Liquid	-0.08	0.18	[-0.43, 0.26]	0.635	
	Combined	0.08	0.18	[-0.26, 0.43]		
Cost per test, ₹	50	0.44	0.20	[0.07, 0.86]	0.012	*
	100	-0.44	0.20	[-0.86, -0.07]		

Table K. Parameter estimates for full conjoint analysis interviews of entire population (main population and local contacts, N = 53), along with the standard error of the estimate, 95% confidence interval, p-value according to a likelihood ratio test, and significance level, where * denotes $p < 0.05$ and ** denotes $p < 0.005$, for each level of each design attribute.

Attribute	Level	Estimate	Std. Error	95% CI	p-value	Significance
Reusability	Disposable	-0.21	0.07	[-0.36, -0.08]	0.0021	**
	Mostly Reusable	0.21	0.07	[0.08, 0.36]		
Output	Amount	-0.62	0.08	[-0.78, -0.47]	<0.0001	**
	Amount + Recommendation	0.62	0.08	[0.47, 0.78]		
Time to results	Next Day	-0.30	0.07	[-0.45, -0.16]	<0.0001	**
	Same Day	0.30	0.07	[0.16, 0.45]		
Ingredients	Add Liquid	-0.18	0.07	[-0.32, -0.05]	0.0078	*
	Combined	0.18	0.07	[0.05, 0.32]		
Cost per test, ₹	50	0.31	0.08	[0.16, 0.46]	<0.0001	**
	100	-0.31	0.08	[-0.46, -0.16]		

8 Conjoint analysis with demographic interaction effects

Considering the effects of participant demographics on preferences for design attributes allows us to identify effects worthy of future investigation for market segmentation and a deeper understanding of water quality management needs in a given resource-limited setting. Evaluation of demographic interactions, also referred to as subject-level interactions, in conjoint analysis yields an estimate of the main effect for each design attribute, as well as estimates of the interaction effect for each demographic attribute considered. Recall that when choosing among multiple options, the total utility U_i of option i is modeled as a linear combination of the marginal utilities u_j for each of the N possible attributes, with study design coefficients $a_{ij} \in \{0, 1\}$ denoting whether attribute j is present in option i:

$$U_i = \sum_{j}^{N} a_{ij} u_j \quad (1)$$

When subject-level interactions, including interactions with demographic parameters such as participants' gender and state of residence, are considered, the marginal utility u_{jk} for participant k is a sum of the marginal utility for the main effect u_{j0} and a linear combination of the demographic interaction effects u_{jl} for each of the D demographic attributes investigated, with demographic coefficients $b_{kl} \in \{0, 1\}$ denoting whether participant k is a member of demographic group l:

$$u_{jk} = u_{j0} + \sum_{l}^{D} b_{kl} u_{jl} \tag{2}$$

As with the marginal utilities for different levels of a design attribute, the interaction effects u_{jl} for different values of a demographic attribute (e.g. male and female for gender) are generally calculated to sum to zero. Therefore, when a design attribute and a demographic attribute each have two levels (e.g. "Mostly Reusable" vs. "Disposable" and "male" vs. "female"), corresponding to $j, l \in \{1, 2\}$, the four resulting demographic interaction effects will be related as $u_{12} = u_{21} = -u_{11} = -u_{22}$. The total utility of option i when considered by participant k, denoted as U_{ik}, will then be given by:

$$U_{ik} = \sum_{j}^{N} a_{ij} \left\{ u_{j0} + \sum_{l}^{D} b_{kl} u_{jl} \right\} \tag{3}$$

Note that the estimate u_{j0} for the main effect of the marginal utility for a given attribute when demographic interactions are considered will not necessarily be equal to the estimate u_j for the marginal utility when demographic interactions are not considered, as the conditional logistic regression will simultaneously calculate the maximum likelihood estimate for the main effects u_{j0} and the demographic interaction effects u_{jl} for each design attribute.

In our preliminary investigation of demographic interaction effects, we considered interactions of all five design attributes with participants' state of residence, gender, recruitment method (group model-building workshop or other), membership in women's SHGs, and community leadership role (Tables L and M). Our conjoint analysis study was not designed to have statistical power for any sub-populations except for residents of each state, and the participants in the two states investigated had significant differences in education, gender, and SHG membership, rendering all calculated demographic interactions as preliminary observations.

Among the statistically significant interaction effects (Table L), the most intriguing were those for test reusability (Fig B). We observed a higher preference for mostly reusable tests in Uttarakhand, consistent with greater

logistical concerns in a mountainous state where transportation is more of a challenge than in Jharkhand. We observed that women had a lower preference for mostly reusable tests than men, but this effect was reduced by SHG membership. Further study is warranted to validate this effect, as well as to investigate potential explanations such as SHG members having increased financial awareness of the benefits of capital investments or anticipating that they would share a mostly reusable sensor with other members of the SHG.

In performing conjoint analysis interviews, it is generally desirable to utilize random sampling of a defined population of interest[3,4]. However, in evaluating the preferences of hard-to-reach populations, such as remote rural residents in resource-limited settings, random sampling is frequently not feasible due to the difficulties in defining a sampling frame, connecting with interview participants, and establishing sufficient rapport with participants to ensure a high quality and quantity of responses[5]. For this reason, research on hard-to-reach populations frequently makes use of sampling techniques such as convenience sampling, purposive sampling, snowball sampling, venue-based sampling, or derived rapport sampling[5–10]. In our study, we used purposive sampling to evaluate the preferences of likely initial users for our intended sensing use cases. We accessed participants through derived rapport from our local partner organizations so as to allow for a greater degree of trust between participants and researchers than would have been possible otherwise, with the majority of participants recruited through group model-building (GMB) workshops simultaneously conducted in order to evaluate system dynamics of water quality management[11].

We found no significant subject-level interaction effects betwen recruitment method (GMB workshop vs. other methods such as door-to-door recruiting) and our investigated design attributes (Table M). Moreover, our evaluated preferences from our conjoint analysis interviews were in agreement with our group design workshops and qualitative KAP interviews, resulting in a high degree of consistency over multiple visits, multiple research methods, and multiple study locations. The non-significant observed subject-level effects due to recruitment method are an aspect of evaluating preferences in rural, resource-limited settings, as fully random sampling methods in these environments would require substantially more time and resources than many sensor researchers will be able to allocate while still being subject to the limitations of sampling hard-to-reach populations described above. In other resource-limited settings, such as urban slums, increased density may result in greater feasibility for random sampling methods such as probability proportional to size sampling[4] or other nonrandom sampling approaches such as venue-based sampling[7–9].

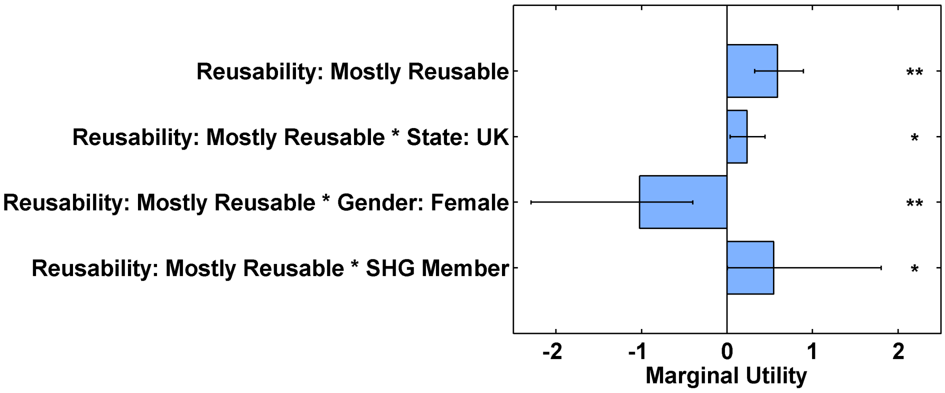

Fig B. Parameter estimates for reusability and corresponding statistically significant ($p < 0.05$ according to likelihood ratio test on each parameter) demographic interactions from the full conjoint analysis interviews of the main population (end users, no local contacts, N = 45) where demographic interactions were considered for respondents' state of residence (Uttarakhand vs. Jharkhand), gender, recruitment method (GMB workshop or other), SHG membership, and community leadership role. Error bars are 95% confidence intervals. Asterisks are significance level, where * denotes $p < 0.05$ and ** denotes $p < 0.005$ according to a likelihood ratio test on each parameter estimate.

Table L. Parameter estimates for statistically significant parameters ($p < 0.05$ according to likelihood ratio test on each parameter) from the full conjoint analysis interviews of the main population (end users, no local contacts, N = 45) where demographic interactions were considered for respondents' state of residence (Uttarakhand vs. Jharkhand), gender, recruitment method (GMB workshop or other), SHG membership, and community leadership role. Significant parameter estimates are presented for each parameter along with the 95% confidence interval (CI), p-value according to a likelihood ratio test, and significance level, where * denotes $p < 0.05$ and ** denotes $p < 0.005$, for each effect.

Attribute	Estimate	95% CI	p-value	Significance
Reusability: Mostly Reusable	0.59	[0.32, 0.89]	<0.0001	**
Output: Amount + Recommendation	0.50	[0.21, 0.82]	0.0003	**
Time to Results: Same Day	0.45	[0.16, 0.80]	0.0011	**
Reusability: Mostly Reusable × State: Uttarakhand	0.23	[0.03, 0.45]	0.0146	*
Reusability: Mostly Reusable × Gender: Female	-1.02	[-2.29, -0.40]	0.0002	**
Reusability: Mostly Reusable × SHG Member	0.55	[0.00, 1.80]	0.0212	*
Time to Results: Same Day × Gender: Female	0.59	[0.03, 1.70]	0.0096	*
Time to Results: Same Day × SHG Member	0.60	[0.11, 1.69]	0.0039	**
Ingredients: Combined × Community Leader	0.24	[0.00, 0.49]	0.0368	*
Cost per Test: ₹50 × Gender: Female	0.61	[0.13, 2.27]	0.0021	**
Cost per Test: ₹50 × SHG Member	0.43	[-0.03, 2.10]	0.0249	**

Table M. Parameter estimates for all parameters from the full conjoint analysis interviews (no local contacts, N = 45) where demographic interactions were considered for respondents' state of residence (Uttarakhand vs. Jharkhand), gender, recruitment method (GMB workshop or other), SHG membership, and community leadership role. Parameter estimates are presented for each parameter along with the 95% confidence interval (CI), p-value according to a likelihood ratio test, and significance level, where * denotes $p < 0.05$ and ** denotes $p < 0.005$, for each effect.

Attribute	Estimate	Std. Error	95% CI	p-value	Significance
Reusability: Mostly Reusable	0.59	0.15	[0.32, 0.89]	$<$0.0001	**
Output: Amount + Recommendation	0.50	0.16	[0.21, 0.82]	0.0003	**
Time to Results: Same Day	0.45	0.16	[0.16, 0.80]	0.0011	**
Ingredients: Add Liquid	-0.04	0.13	[-0.29, 0.23]	1	
Cost per Test: ₹50	0.21	0.17	[-0.10, 0.57]	0.1488	
Reusability: Mostly Reusable × State: Uttarakhand	0.23	0.11	[0.03, 0.45]	0.0146	*
Reusability: Mostly Reusable × Gender: Female	-1.02	0.37	[-2.29, -0.40]	0.0002	**
Reusability: Mostly Reusable × GMB Recruitment	0.27	0.17	[-0.04, 0.61]	0.0651	
Reusability: Mostly Reusable × SHG Member	0.55	0.34	[0.00, 1.80]	0.0212	*
Reusability: Mostly Reusable × Community Leader	0.22	0.13	[-0.02, 0.49]	0.0523	
Output: Amount + Recommendation × State: Uttarakhand	0.19	0.14	[-0.07, 0.47]	0.1343	
Output: Amount + Recommendation × Gender: Female	-0.40	0.27	[-0.91, 0.50]	1	
Output: Amount + Recommendation × GMB Recruitment	0.33	0.18	[-0.01, 0.69]	0.0671	
Output: Amount + Recommendation × SHG Member	0.26	0.26	[-0.64, 0.78]	1	
Output: Amount + Recommendation × Community Leader	-0.23	0.14	[-0.50, 0.05]	0.1596	

Table M – continued from previous page

Attribute	Estimate	Std. Error	95% CI	p-value	Significance
Time to Results: Same Day × State: Uttarakhand	-0.15	0.11	[-0.37, 0.06]	0.1532	
Time to Results: Same Day × Gender: Female	0.59	0.33	[0.03, 1.70]	0.0096	*
Time to Results: Same Day × GMB Recruitment	-0.26	0.16	[-0.61, 0.04]	0.0559	
Time to Results: Same Day × SHG Member	0.60	0.30	[0.11, 1.69]	0.0039	**
Time to Results: Same Day × Community Leader	0.16	0.15	[-0.11, 0.49]	0.1858	
Ingredients: Combined × State: Uttarakhand	0.11	0.11	[-0.10, 0.33]	0.2995	
Ingredients: Combined × Gender: Female	-0.43	0.23	[-0.88, 0.23]	1	
Ingredients: Combined × GMB Recruitment	0.21	0.15	[-0.06, 0.51]	0.0999	
Ingredients: Combined × SHG Member	0.31	0.21	[-0.34, 0.73]	1	
Ingredients: Combined × Community Leader	0.24	0.13	[0.00, 0.49]	0.0368	*
Cost per Test: ₹50 × State: Uttarakhand	-0.04	0.12	[-0.28, 0.20]	0.8966	
Cost per Test: ₹50 × Gender: Female	0.61	0.29	[0.13, 2.27]	0.0021	**
Cost per Test: ₹50 × GMB Recruitment	-0.14	0.15	[-0.47, 0.16]	0.4176	
Cost per Test: ₹50 × SHG Member	0.43	0.29	[-0.03, 2.10]	0.0249	**
Cost per Test: ₹50 × Community Leader	0.01	0.16	[-0.28, 0.34]	1	

References

[1] de Bekker-Grob EW, Donkers B, Jonker MF, Stolk EA. Sample Size Requirements for Discrete-Choice Experiments in Healthcare: a Practical Guide. Patient. 2015;8:373–384.

[2] Bono MS, Beasley SB, Hanhauser EB, Hart AJ, Karnik RN, Vaishnav C. Method for point-of-use testing for bacteriological water contamination in resource-limited environments; Filed Oct. 18, 2016. U.S. Provisional Patent Application No. 62/409,541.

[3] Farber S, Griner B. Using Conjoint Analysis To Value Ecosystem Change. Environmental Science & Technology. 2000;34:1407–1412.

[4] Poulos C, Yang JC, Patil SR, Pattanayak S, Wood S, Goodyear L, et al. Consumer preferences for household water treatment products in Andhra Pradesh, India. Social Science & Medicine. 2012;75:738–746.

[5] Ellard-Gray A, Jeffrey NK, Choubak M, Crann SE. Finding the Hidden Participant: Solutions for Recruiting Hidden, Hard-to-Reach, and Vulnerable Populations. International Journal of Qualitative Methods. 2015;14(5):1–10.

[6] Kinsler JJ, Cunningham WE, na CRN, Nadjat-Haiem C, Grinsztejn B, Casapia M, et al. Using Conjoint Analysis to Measure the Acceptability of Rectal Microbicides Among Men Who Have Sex with Men in Four South American Cities. AIDS and Behavior. 2012;16:1436–1447.

[7] Lee SJ, Newman PA, Comulada WS, Cunningham WE, Duan N. Use of conjoint analysis to assess HIV vaccine acceptability: feasibility of an innovation in the assessment of consumer health-care preferences. International Journal of STD & AIDS. 2012;23(4):235–241.

[8] Muhib FB, Lin LS, Stueve A, Miller RL, Ford WL, Johnson WD, et al. A Venue-Based Method for Sampling Hard-to-Reach Populations. Public Health Reports. 2001;116(1_suppl):216–222.

[9] Camerona MP, Newman PA, Roungprakhon S, Scarpa R. The marginal willingness-to-pay for attributes of a hypothetical HIV vaccine. Vaccine. 2013;31:3712–3717.

[10] Valerio MA, Rodriguez N, Winkler P, Lopez J, Dennison M, Liang Y, et al. Comparing two sampling methods to engage hard-to-reach communities in research priority setting. BMC Medical Research Methodology. 2016;16:146.

[11] Vaishnav C, Beasley S, Bono M, Kothari V, Sharma S, Mallik A. The Evolutionary Dynamics of Indias Rural Water Systems: Part I. In: 35th International Conference of the System Dynamics Society; 2017.
