# Supplementary material for: Fieldwork-based determination of design priorities for point-of-use drinking water quality sensors for use in resource-limited environments
Source: PLoS One. 2020 Jan 24;15(1):e0228140. doi: 10.1371/journal.pone.0228140 (PMC6980542; doi:10.1371/journal.pone.0228140)
Supplement: S1 File — Responses are anonymized and tabulated. (PDF) [file pone.0228140.s002.pdf]

Survey Information	Survey 1	Survey 2	Survey 3	Survey 4	Survey 5	Survey 6 (Group survey, eight people)
1. Date	1/13/2016	1/13/2016	1/13/2016	1/13/2016	1/13/2016	1/25/2016
2. Time	Afternoon	Afternoon	Afternoon	Afternoon 4:37pm	Afternoon	Midday, 11:40am - 1:10 pm
3. Location Name	Taldev, Shedashi, Raigad District,	Shedashi (Tribal area), Raigad District,	Shedashi (Tribal area), Raigad District,	Shedashi, Raigad District, Maharashtra	Shedashi, Raigad District, Maharashtra	PRADAN Office, Torpa, Khunti District,
5. Researcher	Chintan, Ramprasad	Mike	Mike	Sydney	Sydney	Sydney, Mike
6. Translator	Chintan, Ramprasad	Harshal	Harshal	Aniket	Aniket	Mukesh
7. Survey Language	Hindi	Marathi	Marathi	Marathi	Marathi	Hindi
8. Test Demonstration	TDS meter	TDS meter	TDS meter	TDS meter	TDS meter	TDS meter, pH strip
**KAP**						
1. What is your drinking water source?	Ground water (dugwell water). Water pumped to a tank and then piped to public taps outside homes	Open well, 1.5-2 km away.	Community tap, 5-min walk away. Every month pay 100 INR to GP for water			Combination of handpumps and wells
2. Are there any alternative drinking water sources to use?		Participant sometimes mentioned a stream instead.	Well nearby is not in use.			For weddings and other large ceremonies, sometimes have to use dug wells because it's the only source with enough water, even though it has leaves and other debris in it. One time went to village for a wedding, handpump ran dry, so had to drink from open well which tasted like soap and people got sick.
3. How would you rate the cleanliness of your water?	Good	Very satisfied	Very good	The water is good	The water in the open well is better than the borewell. The spring was better and both taste and appearance were better. The water is good. Why? She gets the water very close.	Neutral
4. Why do you think so?	We can tell if the fishes do not die in water; no major illnesses from drinking water	Really happy with taste, no smell	Tastes good, no bad smell, doesn' think that there are any problems in the village			
5. Does your community have problems with water?	No	No	Doesn't think that there are any problems in the village. Everything's fine	Yes, people fall sick. The quality is poor. There are no mechanisms to take out water from the well. Water shortage is not a problem. Note: She gets 2 containers of water everyday. There is a tap that is not operational. Some economically stable people have private borewells. After putting in bleach she has faced no problems. (She doesn't independently put in bleach)	As of now there is no water problem in the village - everyone has a borewell. When she shifted from open to borewell she faced problems because of change. (sickness)	Most villages are drinking unsafe water, city people get clean water from the government.
5A. irregular supply	No	No	Water available year-round, but only in the morning when the pump motor is turned on for the borewell			
5B. water shortages	No	No	No			
5C. poor quality	No	No	No			
5D. poor infrastructure	No	Have to go fetch water	No			
5E. anything else?	We have a well and a water tank for the community with piped water scheme. 7 ft water level in the well, 2 ft extracted every day.	No	No			
6. How do you determine if water is not safe to drink?	By color; with cloth/nylon filter; by adding TCL powder to well every 15 days	Satisfied with water because the source is a stream, flowing fresh water, and tastes good	Taste and smell	It takes a 1/2 hour to reach the water source, which is an open well. Bleaching powder is added to the water. She can see if drinking water is not safe by looking at it.	Her water source is a borewell. 100 R/month per water. She visually examines the water to see if it is safe. If the borewell is not working (no electricity) she has to go to the open well 2km away.	By seeing the clarity and taste of the water. Use bleaching powder to preemptively treat water. No one is using any kind of field test kits. Sometimes find insects in water.
7. Do you treat your drinking water in some way?	Use cloth filter; add 200g of TCL for 10-12 Ft of water	No filtration or treatment, but then said that they add powder [presumably bleaching powder]	No filtration or bleach powder (Harshal is doubtful about this claim)	She uses a cloth/something similar to jali mesh.	When husband came home he took over: No treatment individually... but there are bleach powder and frops from GP to each family. There are health problems in the rainy season and the GP is more active then. Not everyone uses the frops because of the taste.	Use bleaching powder to preemptively treat water.
8. Why did you decide to use these treatment methods? (If applicable)		Person appointed by Gram Sabha told all people in the villages that they should use powder for safety reasons. Get free of cost, go to GP to pick up a packet. When it's just him and his wife, go through a packet once every 2 months. When they have a guest, go through a packet a month. GP told them what proportion to use.	No one has every told them that they should treat the water	People from GM told her to use the mesh (she uses even when it appears clean)		
9. Do you know of any other ways to filter or treat water?						Boil water, mostly during rainy season. Filter with clean cloth over mouth of handpump.

10. Have you every tried to learn about water purification methods?		No	No	No		
11. When faced with a water and sanitation problem in your community, how would (or do) you try to solve it?		Water quality is really good, and he's satisfied with it	No problems	She has never faced water quantity issues. After the rainy season the water gets dirty in a the well so she goes to private borewell. She complains to senior members who have Gram Panchayat connections.	If the water in the well is very dirty a group of people will clean the open well (he has been on that team before)	Sometimes use bleaching powder, clean the well, clear area around the well, or switch to a different source. Don't go to government people, but complain to Jal Sahiya, who doesn't do anything. Sometimes the Asha gives bleaching powder, but sometimes they don't, so residents have to buy it themselves. Don't give bleaching powder at the right time.
12. Does contaminated water cause any problems?	Kidney stones, if drinking from borewell		No			Cold, Jaundice, Pimples (from bathing), Itching, Diarrhea, Teeth get yellow,
13. Who is responsible for water and sanitation in your community?	He is the Jal Surakshak, and adds TCL	Gram Panchayat	GP	There are 3 people in the community that care for water (who are independent of the gvt)	She doesn't know anyone responsible for watsan.	It's the duty of the village Jal Sahiya to add bleaching powder, but she doesn't always do it.
14. Do you know of any water testing in your community?	Every three months GP [Gram Panchayat], PHC [Public Health Center] collect samples to test	People from GP visit water source, but it's erratic. Not sure what they do	No	There are people from (Pen) city that come to test once/month and take a water sample	She does not know of any water testing in community. Husband who came in later: A sales person from some water purifying kits compared Bisleri (bottled water) to regular water through tests.	Jal Sahiya also has a test kit, ordered by block to test water, sends report to block, but block doesn't do anything and Jal Sahiya has no treatment options. One person from PRADAN came to check water for chickens [presumably as part of PRADAN's agriculture work]
**PRODUCT RESPONSE**						
Test Demonstration: TDS test				TDS result: 150	TDS result: 77	
Observation						
1. Did the subject potentially introduce contamination?		No	No			
2. If so, how?		No	No			
3. Did the subject follow the testing protocol?		Eventually yes	Subject wasn't comfortable with doing test			Yes
If not, how?		Press mode button instead of hold button, but did measurement correctly the second time				
Did the subject ask for help?		Didn't ask, but was a bit nervous	Yes			Participants helped each other, but they seemed to get an accurate reading.
If so, how?		We helped him identify the right buttons to push	She said that she's shy and it's difficult for her to use. She wasn't comfortable doing the test, and that the button part is difficulty for her to understand.			
6. Other notes						
**CONCLUSIONS**						
4. Was this test easy or difficult to perform?		Easy	Difficult	She is illiterate so it was difficult "It would be better to have red vs. green colors"	Easy, he could read the numbers and was confident he could do it again. He works in a company and he also helps his wife with farming.	
How easy or difficult?		Very easy	To difficult for her to use. Technology itself is difficult for her to understand			
5. Are you confident that you would be able to perform the test again?		Subject was really confident about pressing buttons and reading a number, but not not the color-coding that explained what the numbers meant	Not at all			
How confident or not confident?		Very confident	Not at all confident			
6A. How would you rate the readibility?		Understood numbers, not color code				
6B. precision?						
6C. usability?						
6D. result time?						
7. Is there anything you would like to tell us about the test?						TDS Electrode: Liked, but what is the remedy? Old people like to say that we have been drinking water a long time, so what is wrong with continuing to drink it? Machine should also show if water is safe, and which contaminants are present (ammonium, etc). pH Strip: Liked better, color change was impressive and easier to understand than TDS electrode. Is ok as-is, would like larger strip. Prefer color change because it's more impressive and interesting.
1. What was the easiest test to use?						
2. What feature was the most difficult to understand?		The color coding				

3. How could you use information from a test like this?		He didn't think that he needs such an instrument. Let GP do their job. Felt very strongly that GP should do it	It's not really required to test because it's good water	She would inform GP with test info. if the water is not drinkable she will treat it at home, She has been trained by other people. Pen people come to conduct workshops.	If he found the impurities, he would put in the drops or powder. He often goes to the farm so he drinks water away from home. He could take an instrument like this to anywhere in order to try a new water source. During the rainy season the water is more contaminated. Even in different seasons the water taste is confident in the borewell.	
4. How often do you think you would want to use a test like this?		At least once a month, preferably every 15 days		Every season water has a different taste. She would only test if there is a change in taste.		
5. What do you think your main obstacles to getting cleaner water are?				The obstacle is high speed winds cause dirt in wells because it is an open surface.		
6. What is your income category?		Ration card color is green	Has never had to migrate, has agricultural land			
7. What would you be willing to pay for a test that could inform you of the water quality?		If there's a cost associated, I won't buy it		As of now the water quality is good, she doesn't know how much she'd pay because she has never had any problems.	No ideas how much he'd pay	
NOTES		TDS reading 128 ppm	TDS reading 82 ppm. Says that we're the first student or NGO people to visit the village		GP doesn't provide any training or workshop.	
					Shedashi has 45 households, 3 households have borewells. 8-10 households ask water from them. 1st borewell was installed 10 years back, and the next 2 were installed last year.	

Notes:
1) All responses are as provided by participants, including contradictory information within a given interview.
2) Government-mandated water quality is initiated at the village (Gram Panchayat, abbreviated as GP) level, with implementation varying by state: In Maharashtra, male village-level officials referred to as Jalsurakshaks collect water samples for testing at local (block-level) laboratories, whereas in Jharkhand the corresponding officials are female, known as Jalsahiyas, and use field test kits for measurement of water pH, iron, fluoride, and nitrate content.
