# Supplementary material for: Fieldwork-based determination of design priorities for point-of-use drinking water quality sensors for use in resource-limited environments
Source: PLoS One. 2020 Jan 24;15(1):e0228140. doi: 10.1371/journal.pone.0228140 (PMC6980542; doi:10.1371/journal.pone.0228140)
Supplement: S2 File — (PDF) [file pone.0228140.s003.pdf]

Interviewer: Translator: Date: Village: TDS reading:

Introduction & Explanation
My name is ________ & I am working on a project at my university. Our project is to study drinking water quality and develop an affordable water quality test. We are interviewing several people to get a better understanding of water quality in various regions and if people are interested in testing water themselves. The interview is voluntary, you can stop at anytime, and the answers will be anonymous.

Are you willing to spend 20 – 30 minutes talking with us? **VERBAL CONFIRMATION**

Opening Question (demographics)

- What is your occupation? ____

Knowledge, Attitudes, & Practices:

1. What is your drinking water source?

2. Are there any other alternative drinking water sources to use?

3. How would you rate the cleanliness of your drinking water [clean (*swachh*), okay (*thik*), polluted (*dooshit*)]?

1☐ Very Bad 2☐ Bad 3☐ Neutral 4☐ Good 4☐ Very Good

4. How would you rate the cleanliness of the drinking water in the rest of your community?

1☐ Very Bad 2☐ Bad 3☐ Neutral 4☐ Good 4☐ Very Good

5. How do you determine if water is not safe to drink?

6. Do you use a filter or treat your water in any way? How?

7. Do you know of any other ways to filter or treat water?

8. If there is a problem with your water quality what would you do? **Follow up**: Have you done this in the past? Has it worked?

Interviewer: Translator: Date: Village: TDS reading:

9. Do you know anyone who got sick because of polluted water? [OR] What problems can contaminated water cause? **Follow up:** Do you know anyone that has experienced these problems?

10. Where do you go for healthcare?

11. Is there a school in this village? If so, up to what standard?

12. Who is responsible for maintaining water in the village? What is their responsibility?

13. Has anyone ever tested your water? Who has tested? How often? (Knowledge)

14. Have you ever tested the quality of your drinking water? **Follow up:** Can you describe your experience?

15. Have you ever received recommendations about treating your water?

Test demonstration: pH Strip ☐ TDS Electrode ☐

Observations

Did the subject need to ask for help? If so, what in particular did she/he ask for help with?

Was she/he able to get an accurate reading?

Product Response

Was this test easy or difficult to perform? Why?

Do you think it would be useful to have a point-of-use testing kit? What would you do differently if you had such a kit?

How often do you feel you should test your water?

Demographics

1. How old are you? 1☐ Under 30 2☐ 31 – 40 3☐ 41 – 50 4☐ over 50 5☐ __________
2. Gender? 1☐ Male 2☐ Female
3. What is the highest level of education you have completed?
4. People (sleeping) in household 1☐ 2☐ 3☐ 4☐ 5☐ 6☐ 7☐ 8☐ 9☐ ______________________

Follow-Up

Do you have any questions for us?
