# Supplementary material for: Fieldwork-based determination of design priorities for point-of-use drinking water quality sensors for use in resource-limited environments
Source: PLoS One. 2020 Jan 24;15(1):e0228140. doi: 10.1371/journal.pone.0228140 (PMC6980542; doi:10.1371/journal.pone.0228140)
Supplement: S3 File — (PDF) [file pone.0228140.s004.pdf]

POINT-OF-USE WATER QUALITY SENSOR WORKSHOP
MIT TATA CENTER – JANUARY 2016

Workshop Information

1. Date: (D/M/Y) ______________________________ 2. Time: ______________

3. Duration: ______________________

3. Location Name: ____

4. GPS: ________________________ N ________________________ E

5. Researcher(s): ____

6. Translator(s): ____

7. NGO/Community Point(s) of Contact: ____

____

7. Workshop Language: [1]Hindi [2]English [3]Marathi [4]Urdu [5]Other _______________

8. Number of female participants: _______ 9. Number of male participants: _______

10. Participant association with water quality (circle all that apply):

NGO Worker(s) Community leader(s) Community water initiative Utility worker(s)

End users Other:________________

11. Other comments on participant demographics:

____

____

____

12. Plan for information sharing and/or further actions with community, NGO(s), or other key stakeholders, as currently known:

____

____

____

____

13. Researcher signature and date:

____

Introduction (large group)

1. Summary of objectives and motivation as explained in this workshop:

____

____

____

2. Responses to "Do you care about the quality of your water? Why?"

____

____

____

3. Responses to "Who here has had their drinking water tested? Who has tested their own drinking water?"

____

____

____

4. Responses to "What would you like to know about your water?"

____

____

____

5. Additional introduction questions and responses:

____

____

____

____

Water test demonstrations and discussion (small groups)

1. Water tests demonstrated in the following order:

pH/TDS Electrode 1☐ 2☐

pH Strip 1☐ 2☐

2. Positive aspects of pH/TDS electrode:

____

____

____

3. Negative aspects of pH/TDS electrode:

____

____

____

4. Positive aspects of pH strip:

____

____

____

5. Negative aspects of pH strip:

____

____

____

Hierarchy Card Responses (small groups)

1. Responsibility/Ownership: _____ Votes for “Owned at household level”

_____ Votes for “Owned at community level”

Notes: ____

2. Output: _____ Votes for “Presence/Absence” _____ Votes for “Amount of contaminant”

_____ Votes for “Amount of contaminant and recommended action”

Notes: ____

3. “Same-day results”: _____ Votes for more expensive but “Same-day results”

_____ Votes for cheaper but results the next day

Notes: ____

4. Durability: _____ Votes for cheaper and “Disposable”

_____ Votes for more expensive but “Reusable”

Notes: ____

5. “No mixing required”: _____ Votes for “Willing to mix chemicals”

_____ Votes for “Not willing to mix chemicals”

Notes: ____

6. Testing frequency: “How often do you think that your water should be tested?”

____

____

____

____

____

Three most important attributes

Votes each for

_____ "Owned at Community Level"

_____ "Owned at household level"

_____ "Tells presence/absence"

_____ "Tells amount of contaminant"

_____ "Tells amount of contaminant and recommended action "

_____ "Same-day results"

_____ "Disposable"

_____ "Reusable"

_____ "No mixing required"

Information and action discussion

1. Responses to "Do you think that it would be useful to have a point-of-use testing kit? What would you do differently if you had such a kit?"

2. Responses to "What would you like to know about your water? How has this changed since the beginning of this workshop"

3. Responses to "Who do you think is responsible for testing your water?"

4. Responses to "What do you do (or would you do) if you knew that your water was contaminated?"

5. Additional information/action questions and responses:

6. Additional workshop discussion or notes:
