# Supplementary material for: Fieldwork-based determination of design priorities for point-of-use drinking water quality sensors for use in resource-limited environments
Source: PLoS One. 2020 Jan 24;15(1):e0228140. doi: 10.1371/journal.pone.0228140 (PMC6980542; doi:10.1371/journal.pone.0228140)
Supplement: S4 File — (PDF) [file pone.0228140.s005.pdf]

POINT-OF-USE WATER QUALITY SENSOR DESIGN SURVEY FOR END USERS
MIT TATA CENTER – JANUARY 2017

1. Date: (D/M/Y) ____________________________ 2. Time: __________________________________
3. Location Name: ____
5. Researcher: _______________________________ 6. Translator: _____________________________
7. NGO/Community Point(s) of Contact: ____
8. Survey Language: [1]☐Hindi [2]☐English [3]☐Gujarati [5]☐Other _____________
9. Participant association with water quality (circle all that apply):
NGO Worker(s) Community leader(s) Community water initiative Utility worker(s)
Women's SHG Member/Leader/Federation Leader End users Other: _______________________________
10. Survey number used ______________ 11. Check here for self-guided survey: ☐

Introduction
My name is ____________ and I am working on a project for the Tata Center at MIT, a university in the United States. Our project is to study drinking water quality and develop an affordable water quality test. We are interviewing several people to get a better understanding of how people manage water quality in various regions and what they would want from an improved water quality test. This interview is completely voluntary, you can stop at any time, and all answers will be anonymous.

Are you willing to spend 30-40 minutes talking with us? **VERBAL CONFIRMATION**

Demographics:

1. What is your occupation? _________________________________ 2. What is your gender? [1] Male [2] Female
3. How old are you? [1] Under 30 [2] 31 – 40 [3] 41 – 50 [4] over 50 [5] _______
4. What is the highest level of education you have completed? _________________________
5. Other demographic information (landholding, distance from road head, etc.):__________________________
____

Introductory Questions:

1. Where does your drinking water come from?

2. Do you care about the quality of your water? Why?

3. How do you determine if water is not safe to drink?

4. What problems can be caused by contaminated water? **Follow up:** Do you know anyone that has experienced these problems?

Conjoint Analysis

We would like to learn about your preferences for a bacterial water test so that we can develop a new water test that will meet your needs. We will show you 8 pairs of hypothetical water tests and ask which one you would be more willing to purchase. These tests will be either disposable or mostly reusable (*examples: disposable pH strips vs mostly reusable glucose meter*); tell either the amount of contaminant or both the amount and a recommended action; give results either the same day or the next day; and require either adding liquid ingredients (*example: pH drops*) or come with the ingredients already combined (*example: pH strips*). A hypothetical cost will be listed for each choice, and the tests are assumed to be identical other than the description on the card. Note that we are looking to develop a new test, and are not actually selling any tests at this time.

1. Choice 1 ☐ Choice 2 ☐ Explanation: ______________________________
   ____
2. Choice 1 ☐ Choice 2 ☐ Explanation: ______________________________
   ____
3. Choice 1 ☐ Choice 2 ☐ Explanation: ______________________________
   ____
4. Choice 1 ☐ Choice 2 ☐ Explanation: ______________________________
   ____
5. Choice 1 ☐ Choice 2 ☐ Explanation: ______________________________
   ____
6. Choice 1 ☐ Choice 2 ☐ Explanation: ______________________________
   ____
7. Choice 1 ☐ Choice 2 ☐ Explanation: ______________________________
   ____
8. Choice 1 ☐ Choice 2 ☐ Explanation: ______________________________
   ____

Concluding questions:

1. What would you like to know about your water?

2. Has anyone ever tested your water? Have you ever tested the quality of your own drinking water?

3. What do you do (or would you do) if you knew that your water was contaminated?

4. Who do you think is responsible for determining if your water is safe to drink?

5. How often do you think that your water should be tested?
