# Supplementary material for: Fieldwork-based determination of design priorities for point-of-use drinking water quality sensors for use in resource-limited environments
Source: PLoS One. 2020 Jan 24;15(1):e0228140. doi: 10.1371/journal.pone.0228140 (PMC6980542; doi:10.1371/journal.pone.0228140)
Supplement: S5 File — (PDF) [file pone.0228140.s006.pdf]

1

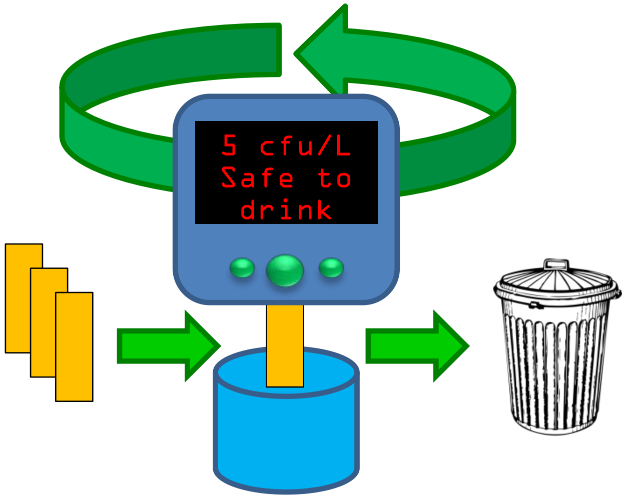

MOSTLY REUSABLE
ज्यादातर पुन: प्रयोज्य

TELLS AMOUNT OF CONTAMINANT AND RECOMMENDED ACTION
दूषित पदार्थों की मात्रा और कार्य योजना बताता है

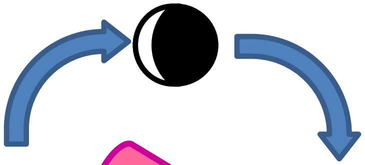

NEXT-DAY RESULTS
अगले दिन परिणाम बतायेगा

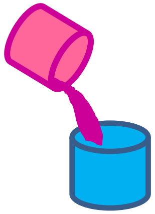

ADD LIQUID INGREDIENTS
तरल सामग्री जोड़ें

REUSABLE PART COST
पुन: प्रयोज्य भाग का मूल्य ₹500

COST PER TEST
प्रति परीक्षण का दाम ₹25

2

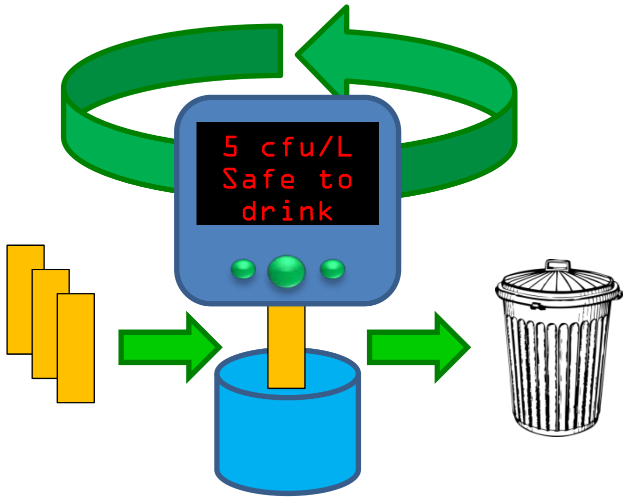

MOSTLY REUSABLE
ज्यादातर पुन: प्रयोज्य

TELLS AMOUNT OF CONTAMINANT AND RECOMMENDED ACTION
दूषित पदार्थों की मात्रा और कार्य योजना बताता है

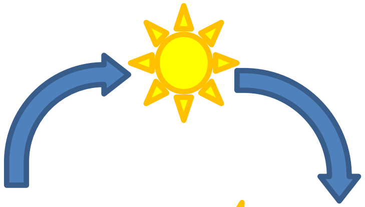

SAME-DAY RESULTS
उसी दिन परिणाम बतायेगा

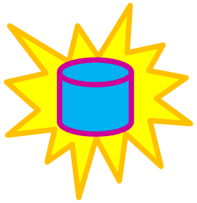

INGREDIENTS ARE ALREADY COMBINED
सामग्री पहले से ही संयुक्त है

REUSABLE PART COST
पुन: प्रयोज्य भाग का मूल्य ₹1000

COST PER TEST
प्रति परीक्षण का दाम ₹50

1

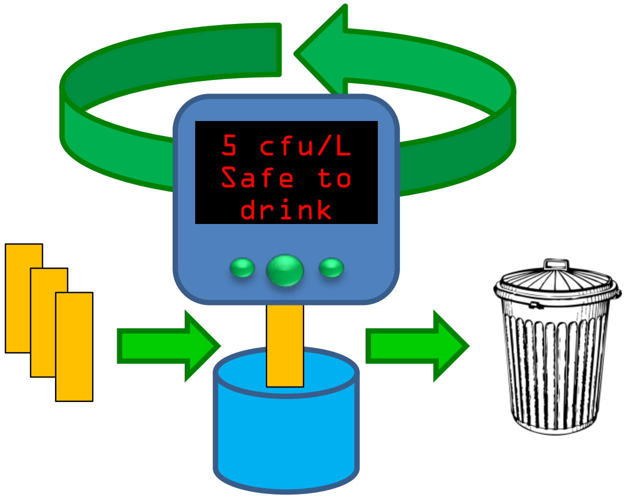

MOSTLY REUSABLE
ज्यादातर पुन: प्रयोज्य

TELLS AMOUNT OF CONTAMINANT AND RECOMMENDED ACTION
दूषित पदार्थों की मात्रा और कार्य योजना बताता है

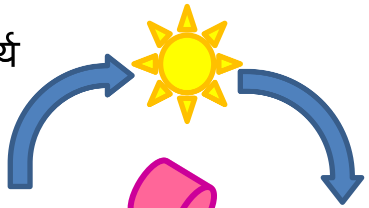

SAME-DAY RESULTS
उसी दिन परिणाम बतायेगा

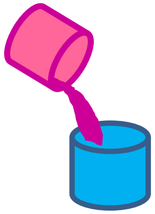

ADD LIQUID INGREDIENTS
तरल सामग्री जोड़ें

REUSABLE PART COST
पुन: प्रयोज्य भाग का मूल्य ₹1000

COST PER TEST
प्रति परीक्षण का दाम ₹50

2

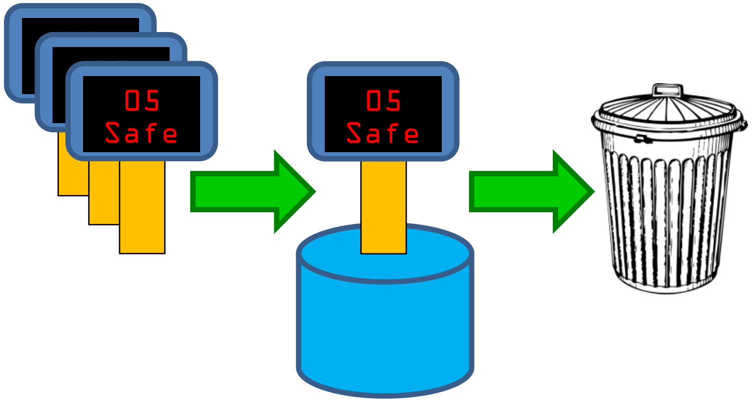

DISPOSABLE
डिस्पोजेबल

TELLS AMOUNT OF CONTAMINANT AND RECOMMENDED ACTION
दूषित पदार्थों की मात्रा और कार्य योजना बताता है

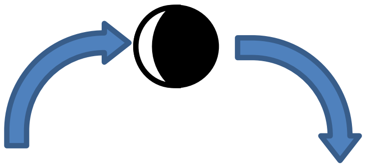

NEXT-DAY RESULTS
अगले दिन परिणाम बतायेगा

INGREDIENTS ARE ALREADY COMBINED
सामग्री पहले से ही संयुक्त है

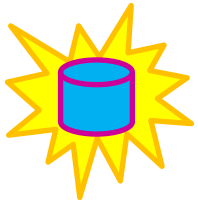

COST PER TEST
प्रति परीक्षण का दाम ₹100

1

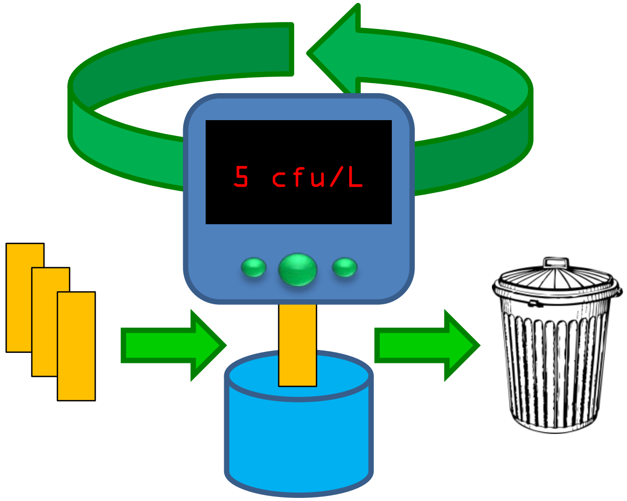

MOSTLY REUSABLE
ज्यादातर पुन: प्रयोज्य

TELLS AMOUNT OF CONTAMINANT
दूषित पदार्थों की मात्रा बताता है

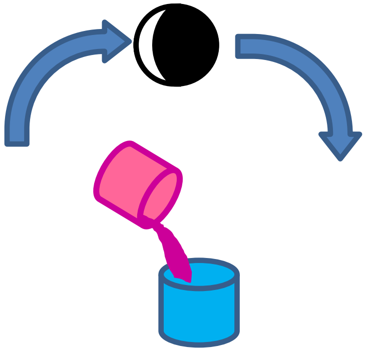

NEXT-DAY RESULTS
अगले दिन परिणाम बतायेगा

ADD LIQUID INGREDIENTS
तरल सामग्री जोड़ें

REUSABLE PART COST
पुन: प्रयोज्य भाग का मूल्य

₹500

COST PER TEST
प्रति परीक्षण का दाम

₹25

2

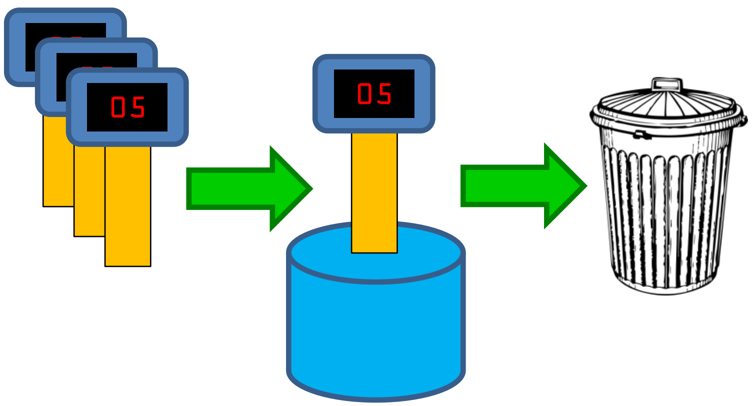

DISPOSABLE
डिस्पोजेबल

TELLS AMOUNT OF CONTAMINANT
दूषित पदार्थों की मात्रा बताता है

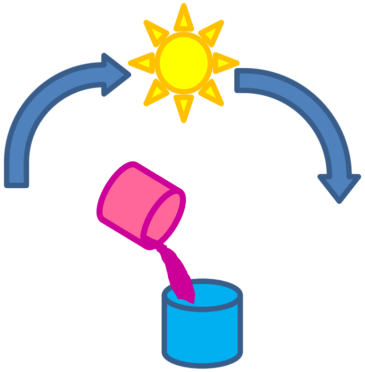

SAME-DAY RESULTS
उसी दिन परिणाम बतायेगा

ADD LIQUID INGREDIENTS
तरल सामग्री जोड़ें

COST PER TEST
प्रति परीक्षण का दाम

₹100

1

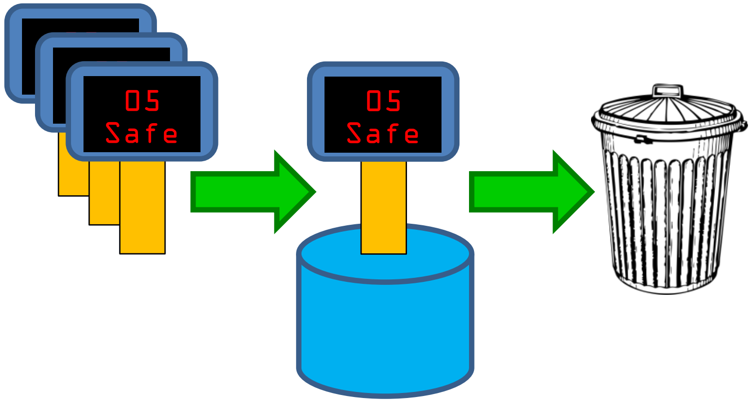

DISPOSABLE
डिस्पोजेबल

TELLS AMOUNT OF CONTAMINANT AND RECOMMENDED ACTION
दूषित पदार्थों की मात्रा और कार्य योजना बताता है

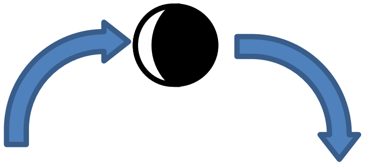

NEXT-DAY RESULTS
अगले दिन परिणाम बतायेगा

INGREDIENTS ARE ALREADY COMBINED
सामग्री पहले से ही संयुक्त है

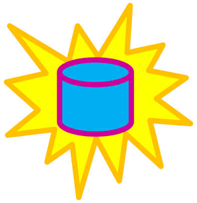

COST PER TEST
प्रति परीक्षण का दाम

₹100

2

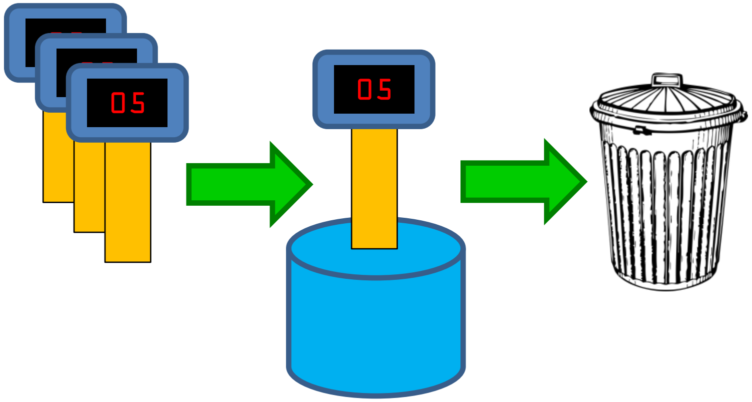

DISPOSABLE
डिस्पोजेबल

TELLS AMOUNT OF CONTAMINANT
दूषित पदार्थों की मात्रा बताता है

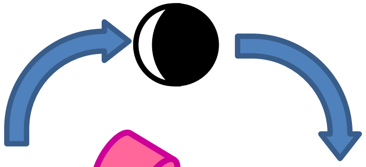

NEXT-DAY RESULTS
अगले दिन परिणाम बतायेगा

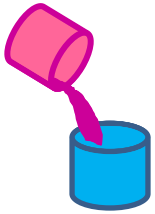

ADD LIQUID INGREDIENTS
तरल सामग्री जोड़ें

COST PER TEST
प्रति परीक्षण का दाम

₹50

1

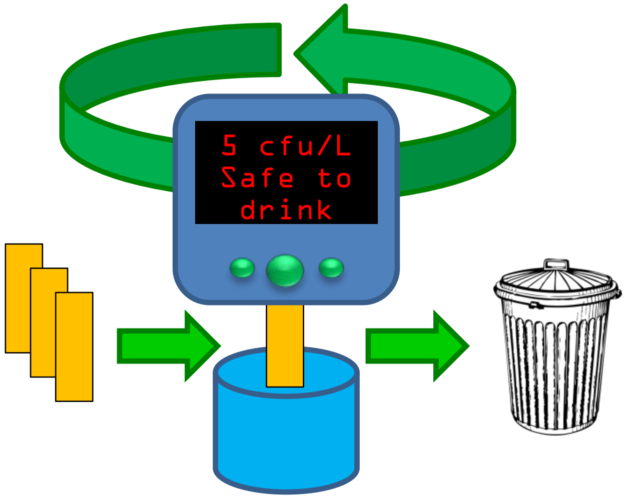

MOSTLY REUSABLE
ज्यादातर पुन: प्रयोज्य

TELLS AMOUNT OF CONTAMINANT AND RECOMMENDED ACTION
दूषित पदार्थों की मात्रा और कार्य योजना बताता है

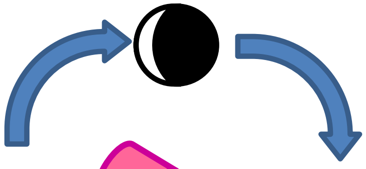

NEXT-DAY RESULTS
अगले दिन परिणाम बतायेगा

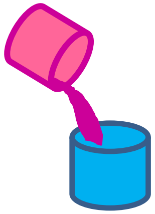

ADD LIQUID INGREDIENTS
तरल सामग्री जोड़ें

REUSABLE PART COST
पुन: प्रयोज्य भाग का मूल्य ₹1000

COST PER TEST
प्रति परीक्षण का दाम ₹50

2

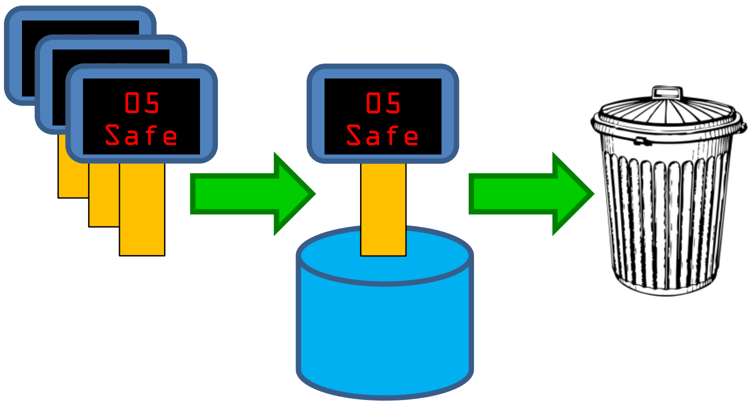

DISPOSABLE
डिस्पोजेबल

TELLS AMOUNT OF CONTAMINANT AND RECOMMENDED ACTION
दूषित पदार्थों की मात्रा और कार्य योजना बताता है

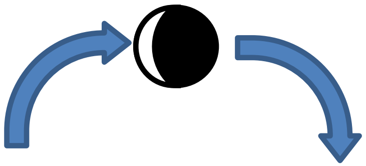

NEXT-DAY RESULTS
अगले दिन परिणाम बतायेगा

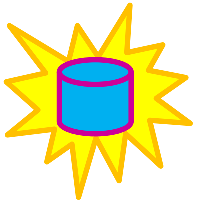

INGREDIENTS ARE ALREADY COMBINED
सामग्री पहले से ही संयुक्त है

COST PER TEST
प्रति परीक्षण का दाम ₹50

1

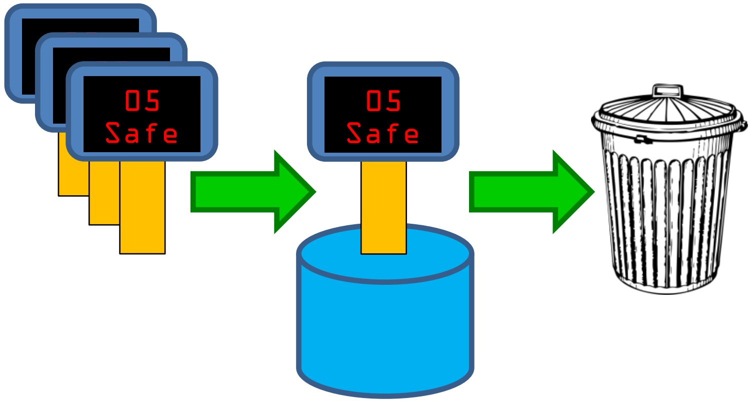

DISPOSABLE
डिस्पोजेबल

TELLS AMOUNT OF CONTAMINANT AND RECOMMENDED ACTION
दूषित पदार्थों की मात्रा और कार्य योजना बताता है

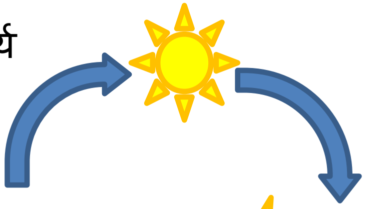

SAME-DAY RESULTS
उसी दिन परिणाम बतायेगा

INGREDIENTS ARE ALREADY COMBINED
सामग्री पहले से ही संयुक्त है

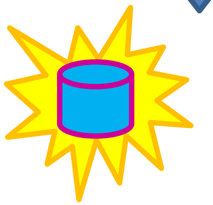

COST PER TEST
प्रति परीक्षण का दाम

₹50

2

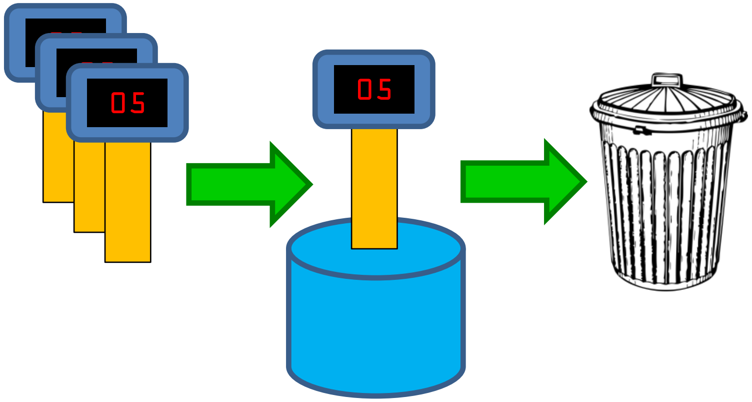

DISPOSABLE
डिस्पोजेबल

TELLS AMOUNT OF CONTAMINANT
दूषित पदार्थों की मात्रा बताता है

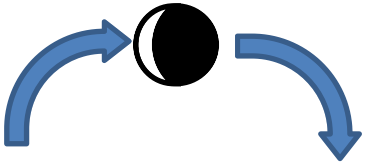

NEXT-DAY RESULTS
अगले दिन परिणाम बतायेगा

INGREDIENTS ARE ALREADY COMBINED
सामग्री पहले से ही संयुक्त है

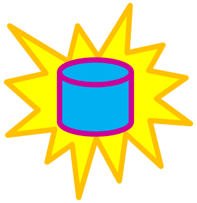

COST PER TEST
प्रति परीक्षण का दाम

₹100

1

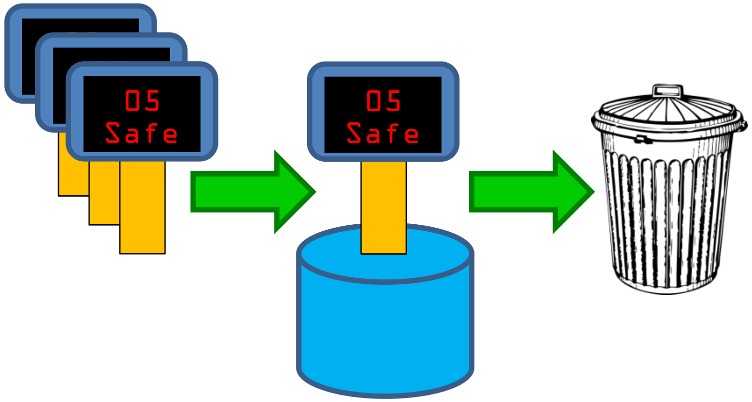

DISPOSABLE
डिस्पोजेबल

TELLS AMOUNT OF CONTAMINANT AND RECOMMENDED ACTION
दूषित पदार्थों की मात्रा और कार्य योजना बताता है

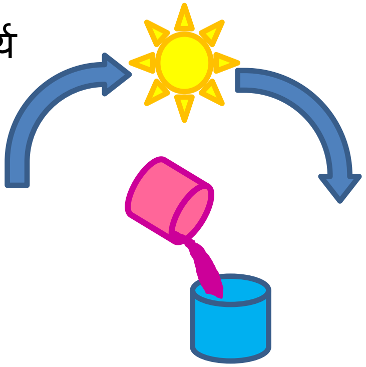

SAME-DAY RESULTS
उसी दिन परिणाम बतायेगा

ADD LIQUID INGREDIENTS
तरल सामग्री जोड़ें

COST PER TEST
प्रति परीक्षण का दाम ₹50

2

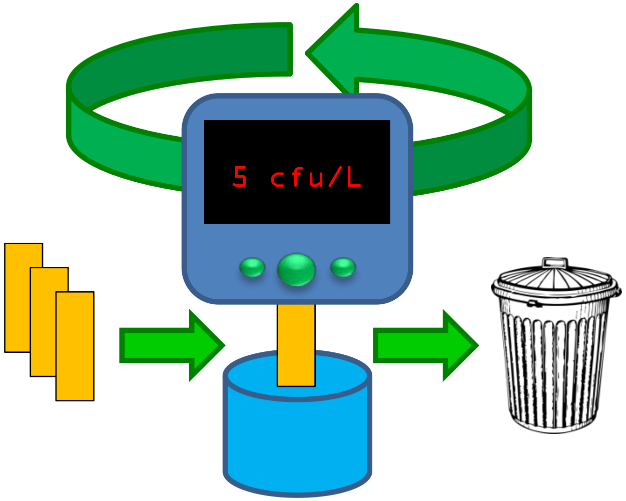

MOSTLY REUSABLE
ज्यादातर पुन: प्रयोज्य

TELLS AMOUNT OF CONTAMINANT
दूषित पदार्थों की मात्रा बताता है

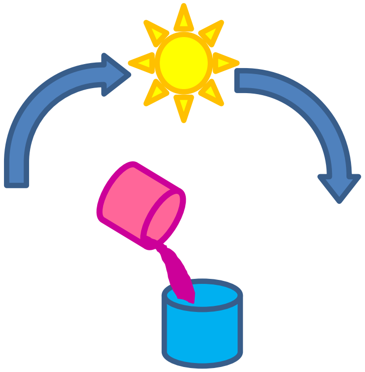

SAME-DAY RESULTS
उसी दिन परिणाम बतायेगा

ADD LIQUID INGREDIENTS
तरल सामग्री जोड़ें

REUSABLE PART COST
पुन: प्रयोज्य भाग का मूल्य ₹1000

COST PER TEST
प्रति परीक्षण का दाम ₹50

1

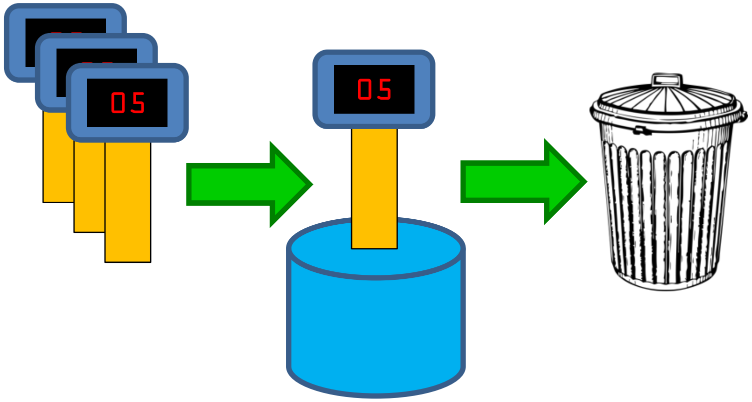

DISPOSABLE
डिस्पोजेबल

TELLS AMOUNT OF CONTAMINANT
दूषित पदार्थों की मात्रा बताता है

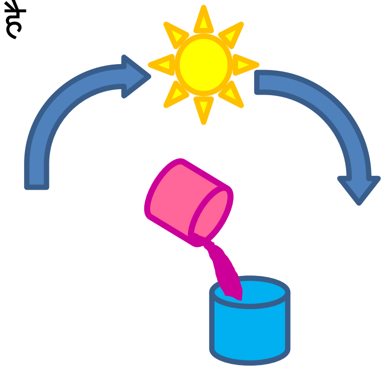

SAME-DAY RESULTS
उसी दिन परिणाम बतायेगा

ADD LIQUID INGREDIENTS
तरल सामग्री जोड़ें

COST PER TEST
प्रति परीक्षण का दाम

₹50

2

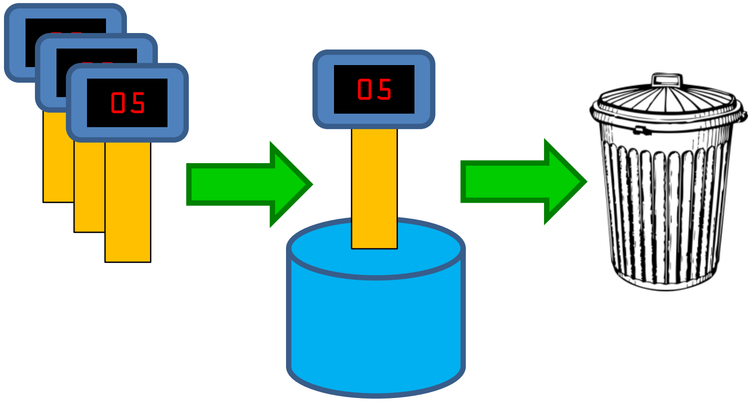

DISPOSABLE
डिस्पोजेबल

TELLS AMOUNT OF CONTAMINANT
दूषित पदार्थों की मात्रा बताता है

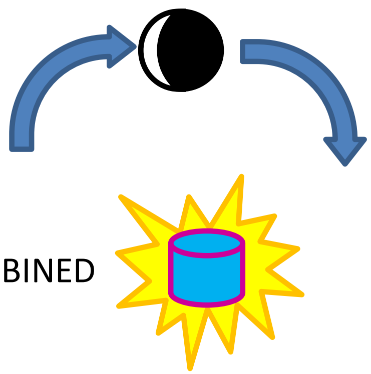

NEXT-DAY RESULTS
अगले दिन परिणाम बतायेगा

INGREDIENTS ARE ALREADY COMBINED
सामग्री पहले से ही संयुक्त है

COST PER TEST
प्रति परीक्षण का दाम

₹100

1

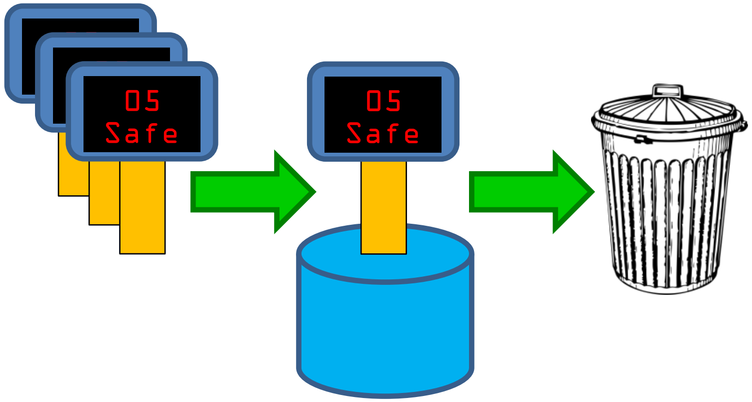

DISPOSABLE
डिस्पोजेबल

TELLS AMOUNT OF CONTAMINANT AND RECOMMENDED ACTION
दूषित पदार्थों की मात्रा और कार्य योजना बताता है

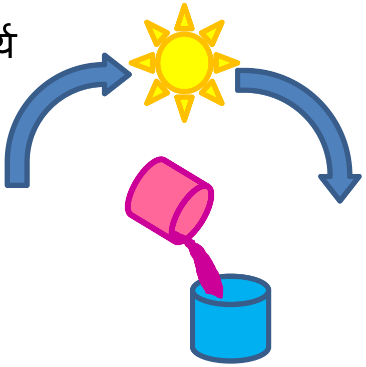

SAME-DAY RESULTS
उसी दिन परिणाम बतायेगा

ADD LIQUID INGREDIENTS
तरल सामग्री जोड़ें

COST PER TEST
प्रति परीक्षण का दाम
₹100

2

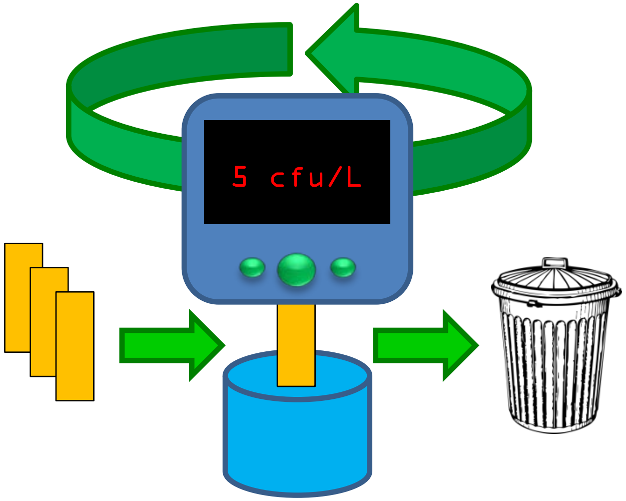

MOSTLY REUSABLE
ज्यादातर पुन: प्रयोज्य

TELLS AMOUNT OF CONTAMINANT
दूषित पदार्थों की मात्रा बताता है

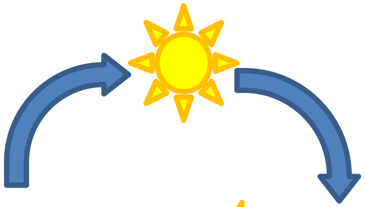

SAME-DAY RESULTS
उसी दिन परिणाम बतायेगा

INGREDIENTS ARE ALREADY COMBINED
सामग्री पहले से ही संयुक्त है

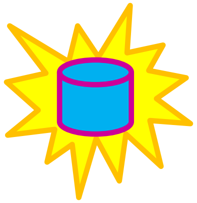

REUSABLE PART COST
पुन: प्रयोज्य भाग का मूल्य
₹1000

COST PER TEST
प्रति परीक्षण का दाम
₹50

1

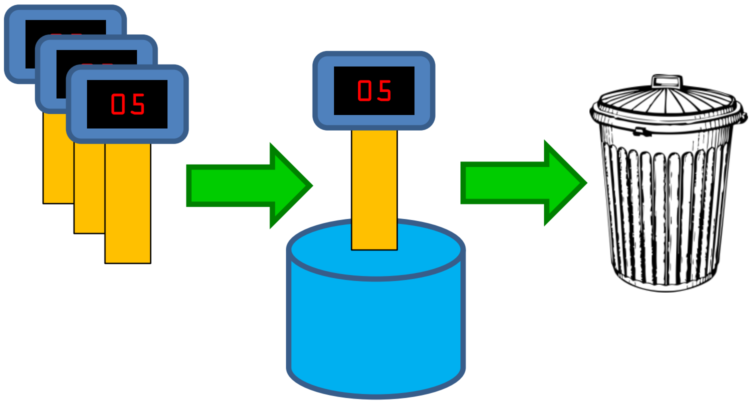

DISPOSABLE
डिस्पोजेबल

TELLS AMOUNT OF CONTAMINANT
दूषित पदार्थों की मात्रा बताता है

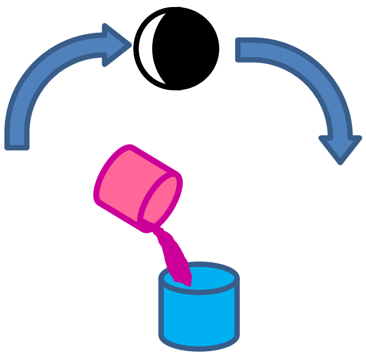

NEXT-DAY RESULTS
अगले दिन परिणाम बतायेगा

ADD LIQUID INGREDIENTS
तरल सामग्री जोड़ें

COST PER TEST
प्रति परीक्षण का दाम

₹50

2

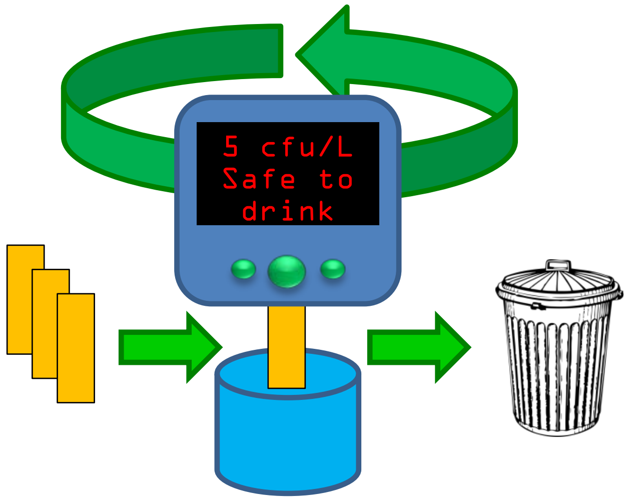

MOSTLY REUSABLE
ज्यादातर पुन: प्रयोज्य

TELLS AMOUNT OF CONTAMINANT AND RECOMMENDED ACTION
दूषित पदार्थों की मात्रा और कार्य योजना बताता है

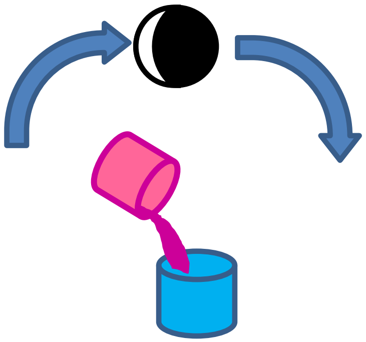

NEXT-DAY RESULTS
अगले दिन परिणाम बतायेगा

ADD LIQUID INGREDIENTS
तरल सामग्री जोड़ें

REUSABLE PART COST
पुन: प्रयोज्य भाग का मूल्य

₹1000

COST PER TEST
प्रति परीक्षण का दाम

₹50

1

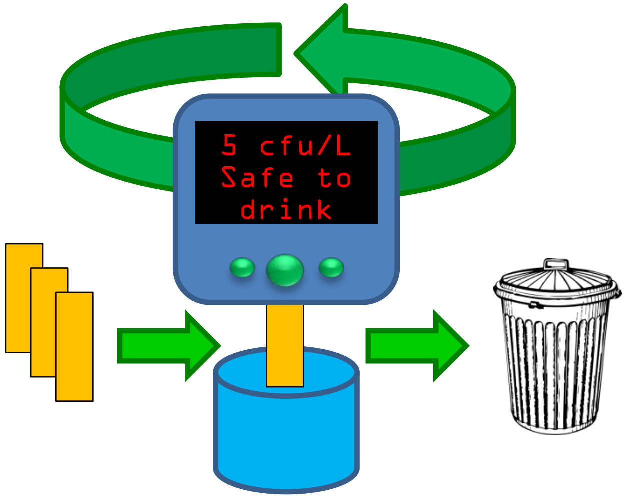

MOSTLY REUSABLE
ज्यादातर पुन: प्रयोज्य

TELLS AMOUNT OF CONTAMINANT AND RECOMMENDED ACTION
दूषित पदार्थों की मात्रा और कार्य योजना बताता है

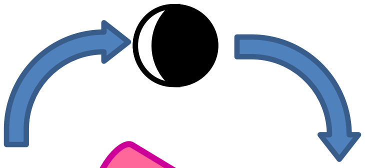

NEXT-DAY RESULTS
अगले दिन परिणाम बतायेगा

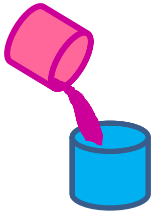

ADD LIQUID INGREDIENTS
तरल सामग्री जोड़ें

REUSABLE PART COST
पुन: प्रयोज्य भाग का मूल्य ₹500

COST PER TEST
प्रति परीक्षण का दाम ₹25

2

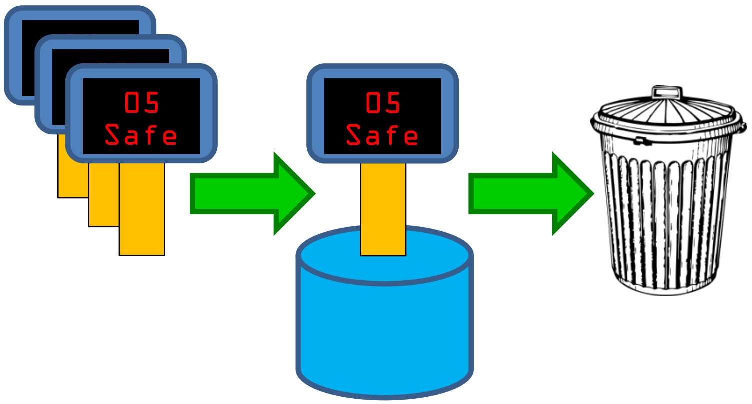

DISPOSABLE
डिस्पोजेबल

TELLS AMOUNT OF CONTAMINANT AND RECOMMENDED ACTION
दूषित पदार्थों की मात्रा और कार्य योजना बताता है

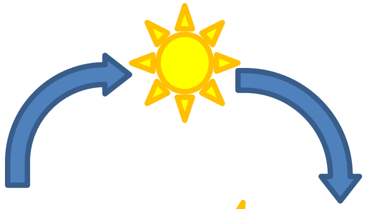

SAME-DAY RESULTS
उसी दिन परिणाम बतायेगा

INGREDIENTS ARE ALREADY COMBINED
सामग्री पहले से ही संयुक्त है

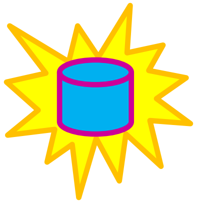

COST PER TEST
प्रति परीक्षण का दाम ₹50

1

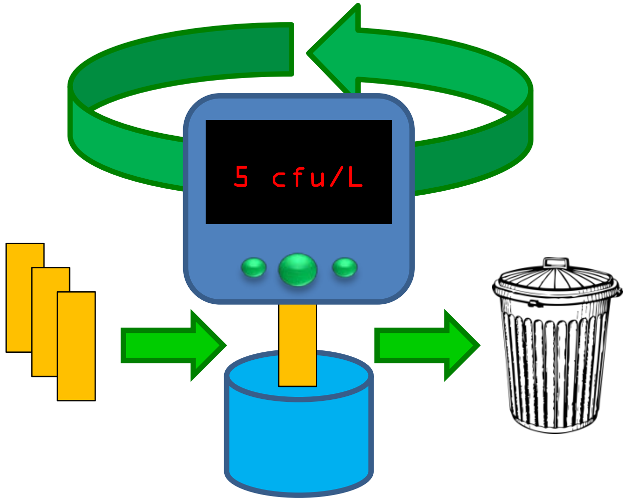

MOSTLY REUSABLE
ज्यादातर पुन: प्रयोज्य

TELLS AMOUNT OF CONTAMINANT
दूषित पदार्थों की मात्रा बताता है

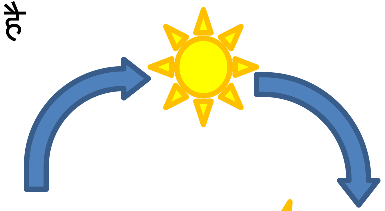

SAME-DAY RESULTS
उसी दिन परिणाम बतायेगा

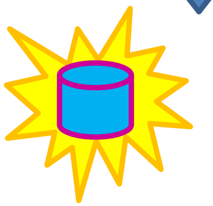

INGREDIENTS ARE ALREADY COMBINED
सामग्री पहले से ही संयुक्त है

REUSABLE PART COST
पुन: प्रयोज्य भाग का मूल्य ₹500

COST PER TEST
प्रति परीक्षण का दाम ₹25

2

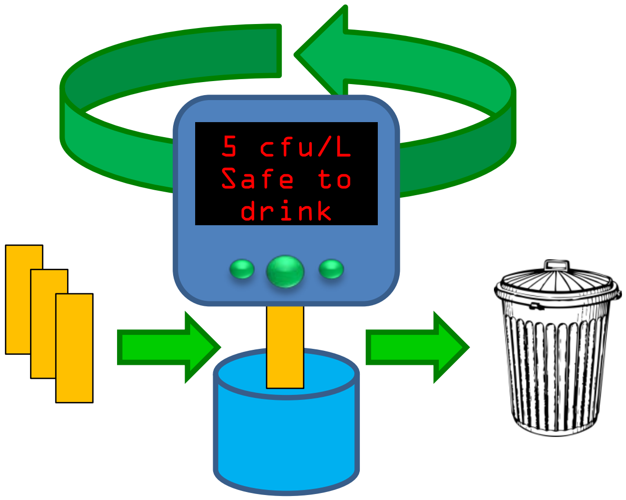

MOSTLY REUSABLE
ज्यादातर पुन: प्रयोज्य

TELLS AMOUNT OF CONTAMINANT
AND RECOMMENDED ACTION
दूषित पदार्थों की मात्रा और कार्य
योजना बताता है

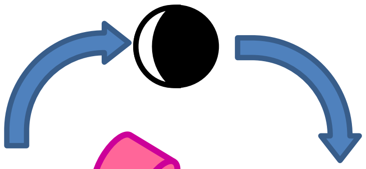

NEXT-DAY RESULTS
अगले दिन परिणाम बतायेगा

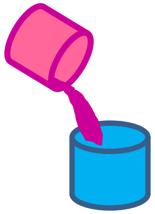

ADD LIQUID INGREDIENTS
तरल सामग्री जोड़ें

REUSABLE PART COST
पुन: प्रयोज्य भाग का मूल्य ₹500

COST PER TEST
प्रति परीक्षण का दाम ₹25

1

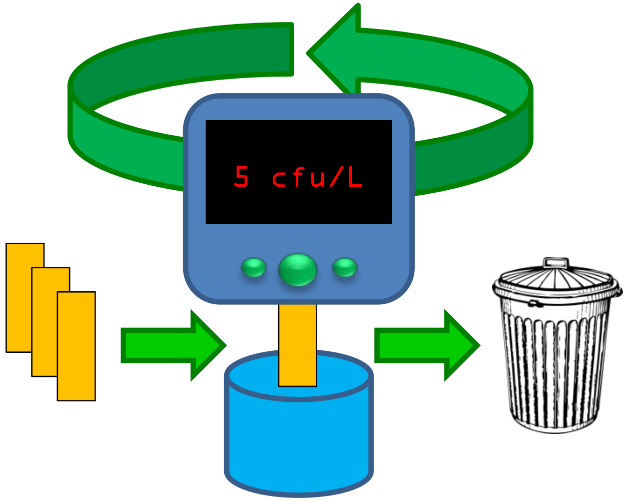

MOSTLY REUSABLE
ज्यादातर पुन: प्रयोज्य

TELLS AMOUNT OF CONTAMINANT
दूषित पदार्थों की मात्रा बताता है

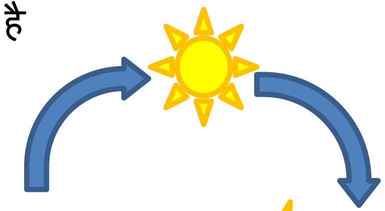

SAME-DAY RESULTS
उसी दिन परिणाम बतायेगा

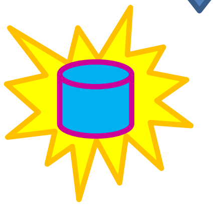

INGREDIENTS ARE ALREADY COMBINED
सामग्री पहले से ही संयुक्त है

REUSABLE PART COST
पुन: प्रयोज्य भाग का मूल्य ₹1000

COST PER TEST
प्रति परीक्षण का दाम ₹50

2

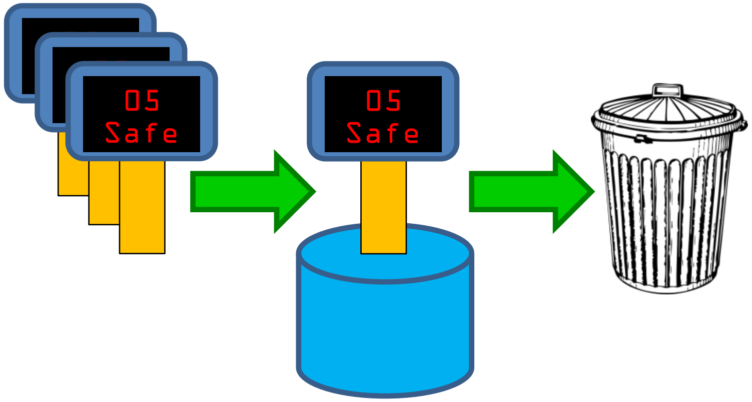

DISPOSABLE
डिस्पोजेबल

TELLS AMOUNT OF CONTAMINANT AND RECOMMENDED ACTION
दूषित पदार्थों की मात्रा और कार्य योजना बताता है

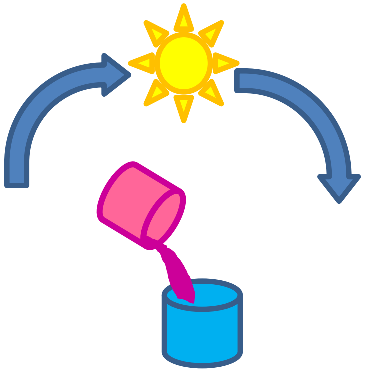

SAME-DAY RESULTS
उसी दिन परिणाम बतायेगा

ADD LIQUID INGREDIENTS
तरल सामग्री जोड़ें

COST PER TEST
प्रति परीक्षण का दाम ₹100

1

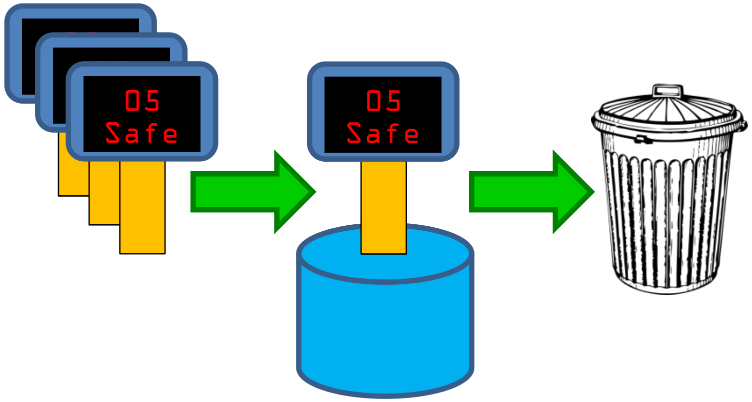

DISPOSABLE
डिस्पोजेबल

TELLS AMOUNT OF CONTAMINANT AND RECOMMENDED ACTION
दूषित पदार्थों की मात्रा और कार्य योजना बताता है

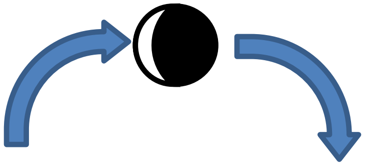

NEXT-DAY RESULTS
अगले दिन परिणाम बतायेगा

INGREDIENTS ARE ALREADY COMBINED
सामग्री पहले से ही संयुक्त है

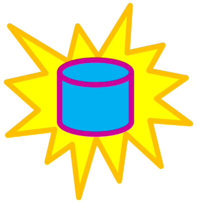

COST PER TEST
प्रति परीक्षण का दाम ₹50

2

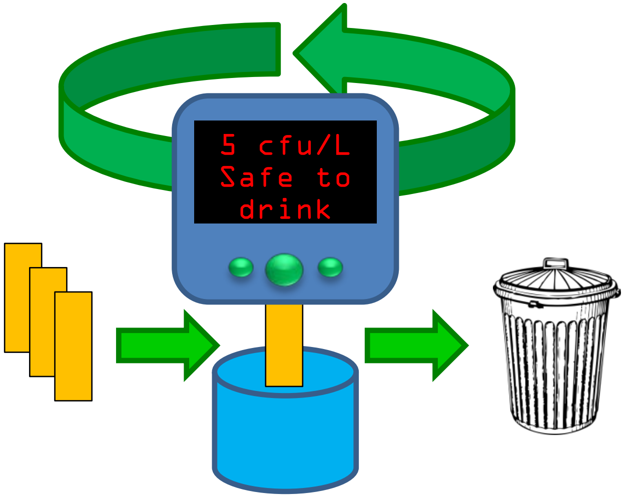

MOSTLY REUSABLE
ज्यादातर पुन: प्रयोज्य

TELLS AMOUNT OF CONTAMINANT AND RECOMMENDED ACTION
दूषित पदार्थों की मात्रा और कार्य योजना बताता है

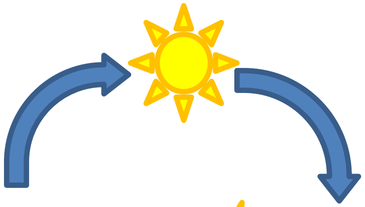

SAME-DAY RESULTS
उसी दिन परिणाम बतायेगा

INGREDIENTS ARE ALREADY COMBINED
सामग्री पहले से ही संयुक्त है

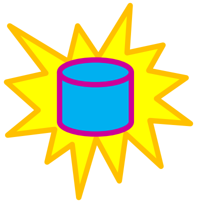

REUSABLE PART COST
पुन: प्रयोज्य भाग का मूल्य ₹1000

COST PER TEST
प्रति परीक्षण का दाम ₹50

1

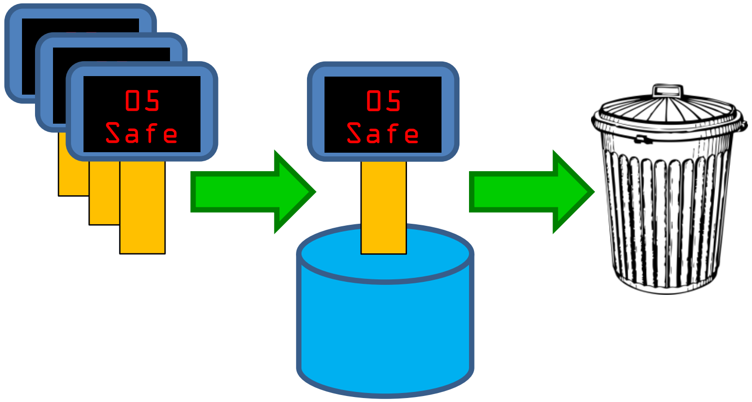

DISPOSABLE
डिस्पोजेबल

TELLS AMOUNT OF CONTAMINANT AND RECOMMENDED ACTION
दूषित पदार्थों की मात्रा और कार्य योजना बताता है

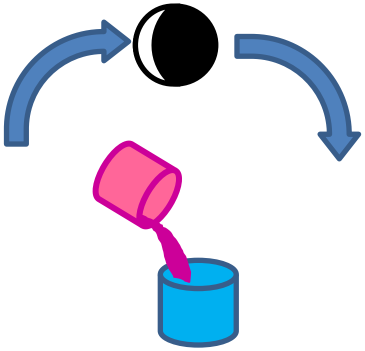

NEXT-DAY RESULTS
अगले दिन परिणाम बतायेगा

ADD LIQUID INGREDIENTS
तरल सामग्री जोड़ें

COST PER TEST
प्रति परीक्षण का दाम

₹100

2

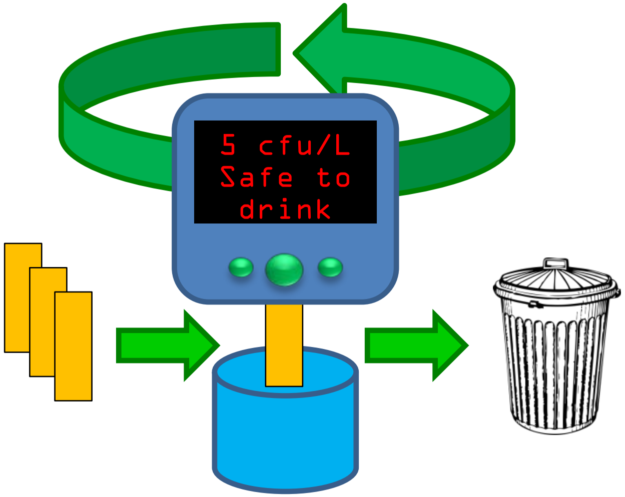

MOSTLY REUSABLE
ज्यादातर पुन: प्रयोज्य

TELLS AMOUNT OF CONTAMINANT AND RECOMMENDED ACTION
दूषित पदार्थों की मात्रा और कार्य योजना बताता है

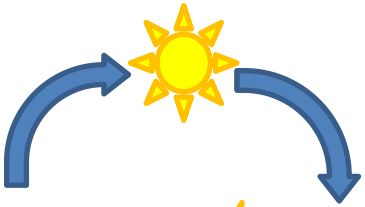

SAME-DAY RESULTS
उसी दिन परिणाम बतायेगा

INGREDIENTS ARE ALREADY COMBINED
सामग्री पहले से ही संयुक्त है

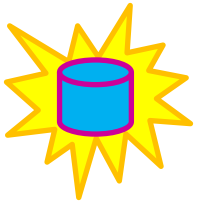

REUSABLE PART COST
पुन: प्रयोज्य भाग का मूल्य

₹1000

COST PER TEST
प्रति परीक्षण का दाम

₹50

1

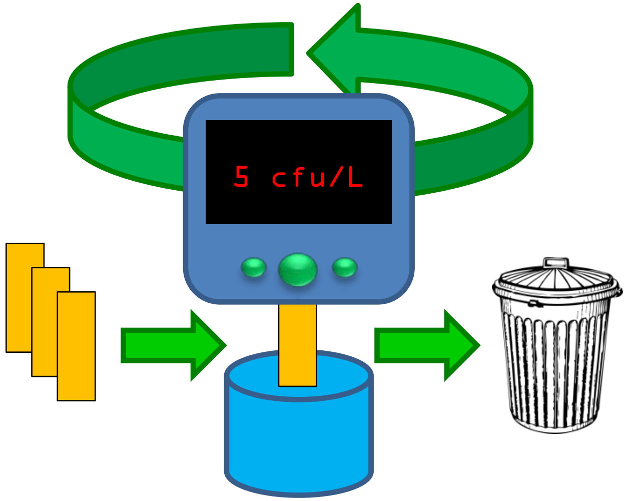

MOSTLY REUSABLE
ज्यादातर पुन: प्रयोज्य

TELLS AMOUNT OF CONTAMINANT
दूषित पदार्थों की मात्रा बताता है

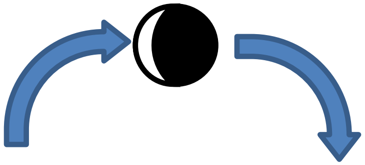

NEXT-DAY RESULTS
अगले दिन परिणाम बतायेगा

INGREDIENTS ARE ALREADY COMBINED
सामग्री पहले से ही संयुक्त है

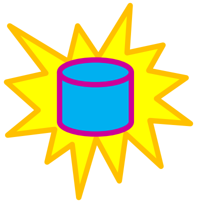

REUSABLE PART COST
पुन: प्रयोज्य भाग का मूल्य ₹500

COST PER TEST
प्रति परीक्षण का दाम ₹25

2

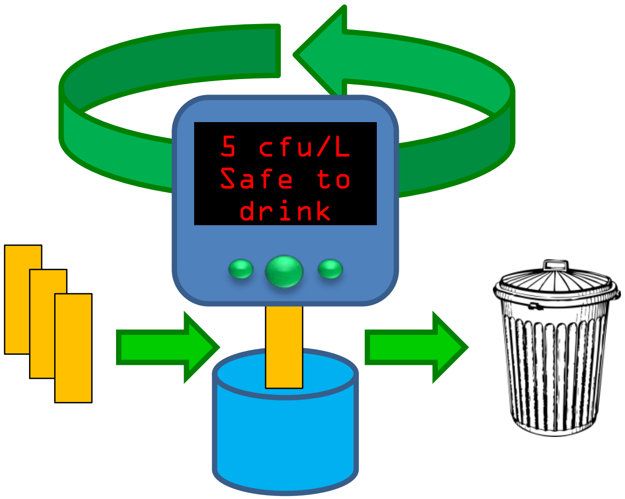

MOSTLY REUSABLE
ज्यादातर पुन: प्रयोज्य

TELLS AMOUNT OF CONTAMINANT
AND RECOMMENDED ACTION
दूषित पदार्थों की मात्रा और कार्य योजना बताता है

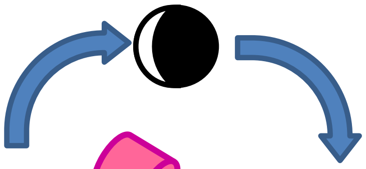

NEXT-DAY RESULTS
अगले दिन परिणाम बतायेगा

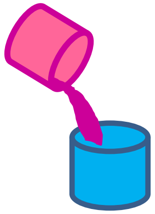

ADD LIQUID INGREDIENTS
तरल सामग्री जोड़ें

REUSABLE PART COST
पुन: प्रयोज्य भाग का मूल्य ₹1000

COST PER TEST
प्रति परीक्षण का दाम ₹50

1

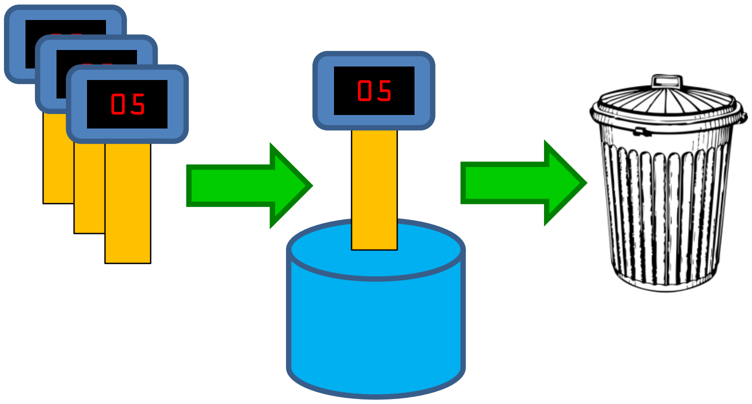

DISPOSABLE
डिस्पोजेबल

TELLS AMOUNT OF CONTAMINANT
दूषित पदार्थों की मात्रा बताता है

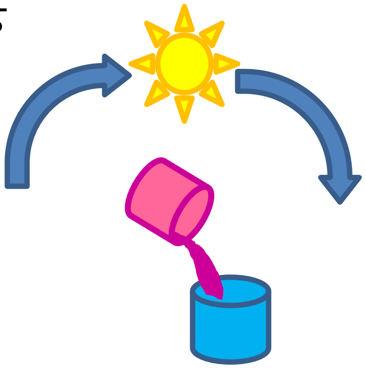

SAME-DAY RESULTS
उसी दिन परिणाम बतायेगा

ADD LIQUID INGREDIENTS
तरल सामग्री जोड़ें

COST PER TEST
प्रति परीक्षण का दाम

₹100

2

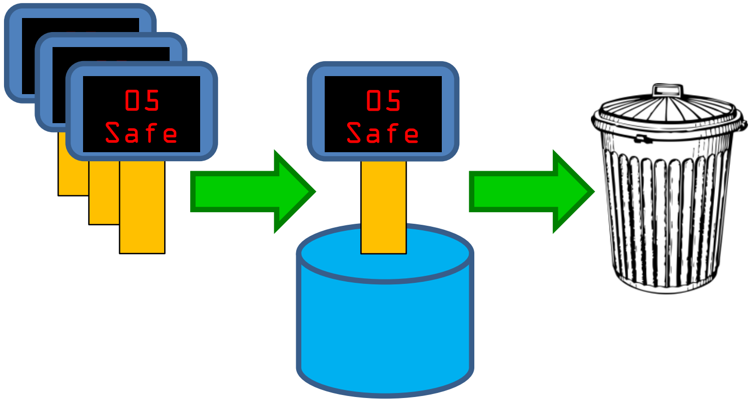

DISPOSABLE
डिस्पोजेबल

TELLS AMOUNT OF CONTAMINANT
AND RECOMMENDED ACTION
दूषित पदार्थों की मात्रा और कार्य
योजना बताता है

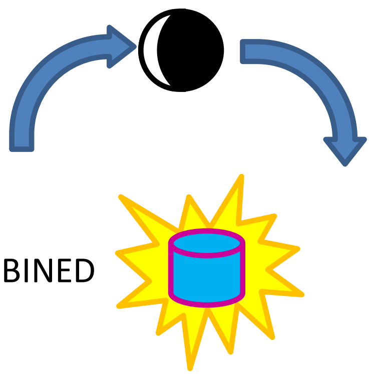

NEXT-DAY RESULTS
अगले दिन परिणाम बतायेगा

INGREDIENTS ARE ALREADY COMBINED
सामग्री पहले से ही संयुक्त है

COST PER TEST
प्रति परीक्षण का दाम

₹100

1

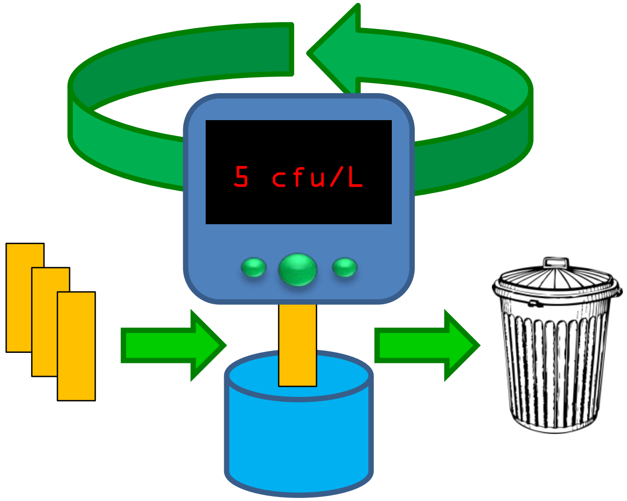

MOSTLY REUSABLE
ज्यादातर पुन: प्रयोज्य

TELLS AMOUNT OF CONTAMINANT
दूषित पदार्थों की मात्रा बताता है

SAME-DAY RESULTS
उसी दिन परिणाम बतायेगा

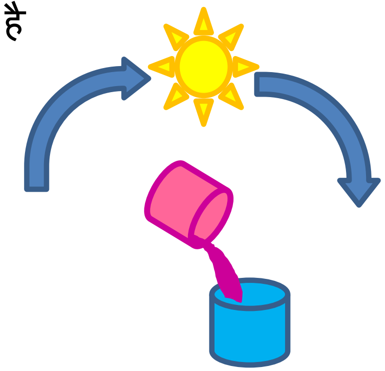

ADD LIQUID INGREDIENTS
तरल सामग्री जोड़ें

REUSABLE PART COST
पुन: प्रयोज्य भाग का मूल्य ₹500

COST PER TEST
प्रति परीक्षण का दाम ₹25

2

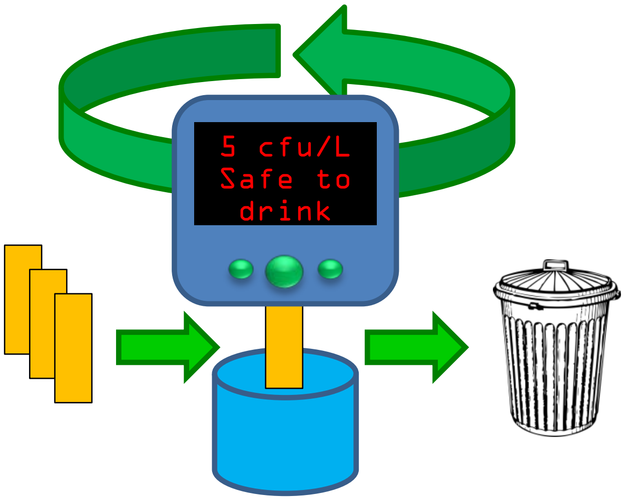

MOSTLY REUSABLE
ज्यादातर पुन: प्रयोज्य

TELLS AMOUNT OF CONTAMINANT AND RECOMMENDED ACTION
दूषित पदार्थों की मात्रा और कार्य योजना बताता है

NEXT-DAY RESULTS
अगले दिन परिणाम बतायेगा

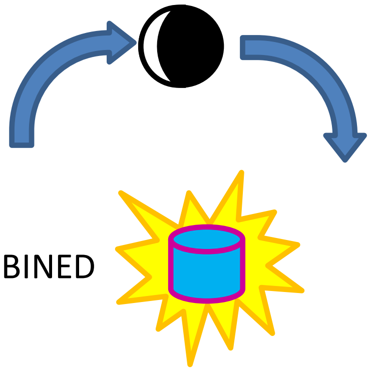

INGREDIENTS ARE ALREADY COMBINED
सामग्री पहले से ही संयुक्त है

REUSABLE PART COST
पुन: प्रयोज्य भाग का मूल्य ₹500

COST PER TEST
प्रति परीक्षण का दाम ₹25

1

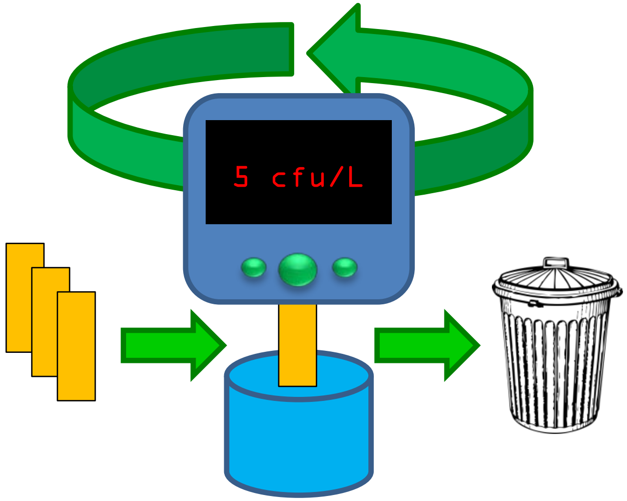

MOSTLY REUSABLE
ज्यादातर पुन: प्रयोज्य

TELLS AMOUNT OF CONTAMINANT
दूषित पदार्थों की मात्रा बताता है

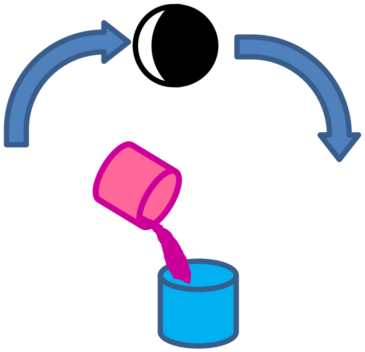

NEXT-DAY RESULTS
अगले दिन परिणाम बतायेगा

ADD LIQUID INGREDIENTS
तरल सामग्री जोड़ें

REUSABLE PART COST
पुन: प्रयोज्य भाग का मूल्य ₹1000

COST PER TEST
प्रति परीक्षण का दाम ₹50

2

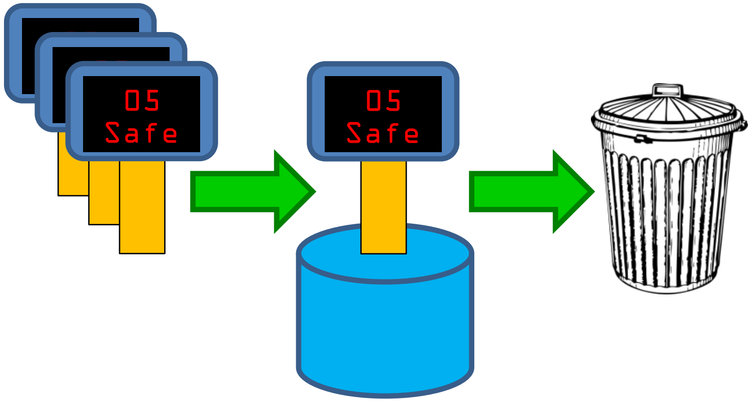

DISPOSABLE
डिस्पोजेबल

TELLS AMOUNT OF CONTAMINANT
AND RECOMMENDED ACTION
दूषित पदार्थों की मात्रा और कार्य
योजना बताता है

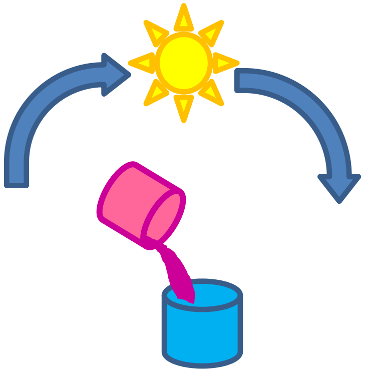

SAME-DAY RESULTS
उसी दिन परिणाम बतायेगा

ADD LIQUID INGREDIENTS
तरल सामग्री जोड़ें

COST PER TEST
प्रति परीक्षण का दाम ₹100

1

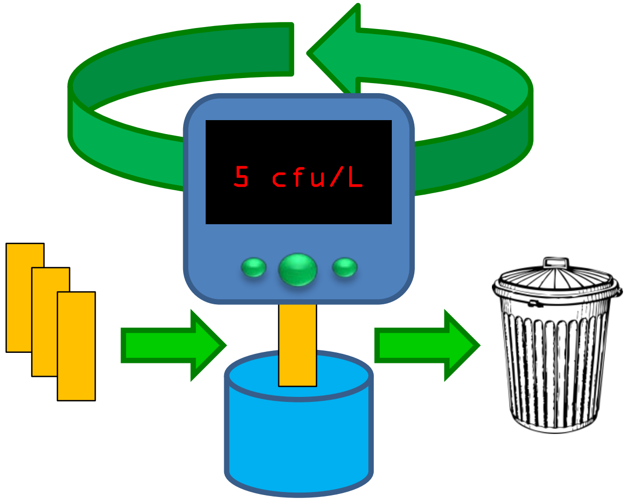

MOSTLY REUSABLE
ज्यादातर पुन: प्रयोज्य

TELLS AMOUNT OF CONTAMINANT
दूषित पदार्थों की मात्रा बताता है

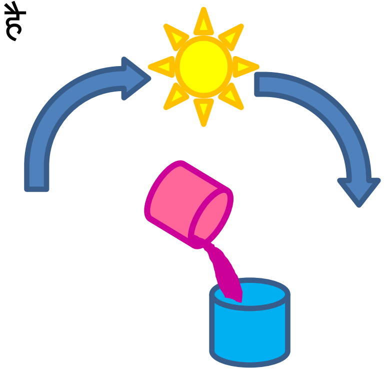

SAME-DAY RESULTS
उसी दिन परिणाम बतायेगा

ADD LIQUID INGREDIENTS
तरल सामग्री जोड़ें

REUSABLE PART COST
पुन: प्रयोज्य भाग का मूल्य ₹1000

COST PER TEST
प्रति परीक्षण का दाम ₹50

2

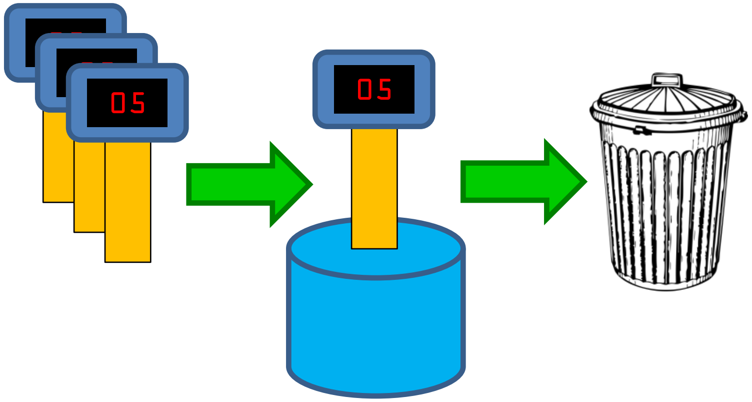

DISPOSABLE
डिस्पोजेबल

TELLS AMOUNT OF CONTAMINANT
दूषित पदार्थों की मात्रा बताता है

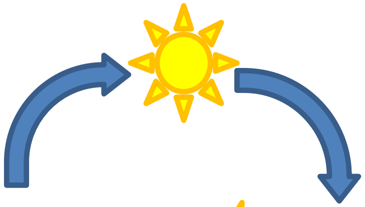

SAME-DAY RESULTS
उसी दिन परिणाम बतायेगा

INGREDIENTS ARE ALREADY COMBINED
सामग्री पहले से ही संयुक्त है

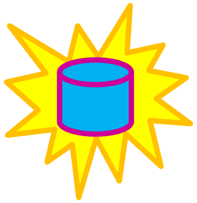

COST PER TEST
प्रति परीक्षण का दाम ₹50

1

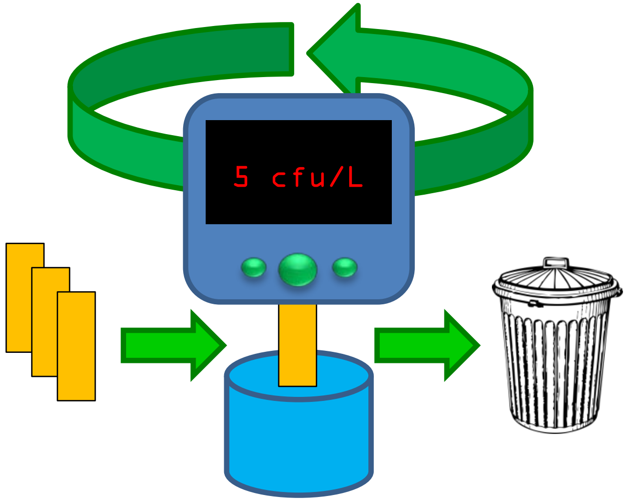

MOSTLY REUSABLE
ज्यादातर पुन: प्रयोज्य

TELLS AMOUNT OF CONTAMINANT
दूषित पदार्थों की मात्रा बताता है

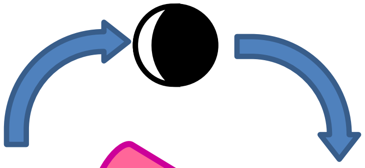

NEXT-DAY RESULTS
अगले दिन परिणाम बतायेगा

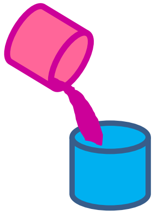

ADD LIQUID INGREDIENTS
तरल सामग्री जोड़ें

REUSABLE PART COST
पुन: प्रयोज्य भाग का मूल्य ₹1000

COST PER TEST
प्रति परीक्षण का दाम ₹50

2

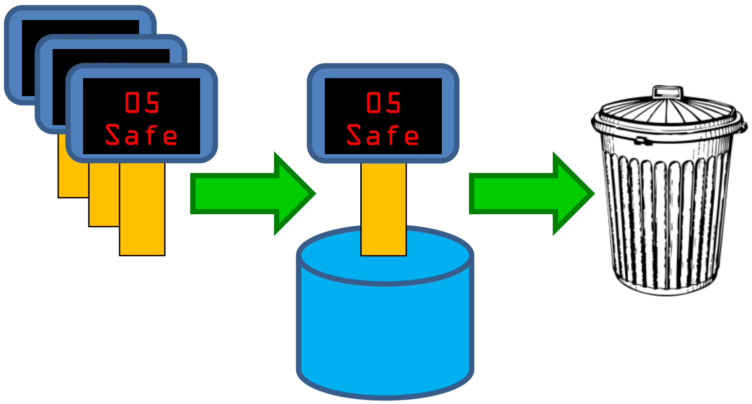

DISPOSABLE
डिस्पोजेबल

TELLS AMOUNT OF CONTAMINANT
AND RECOMMENDED ACTION
दूषित पदार्थों की मात्रा और कार्य योजना बताता है

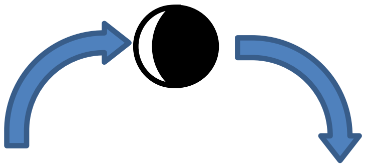

NEXT-DAY RESULTS
अगले दिन परिणाम बतायेगा

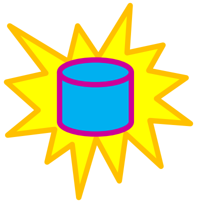

INGREDIENTS ARE ALREADY COMBINED
सामग्री पहले से ही संयुक्त है

COST PER TEST
प्रति परीक्षण का दाम ₹100

1

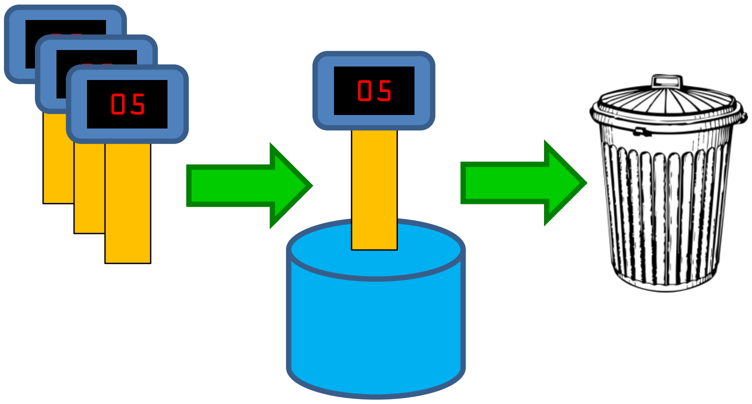

DISPOSABLE
डिस्पोजेबल

TELLS AMOUNT OF CONTAMINANT
दूषित पदार्थों की मात्रा बताता है

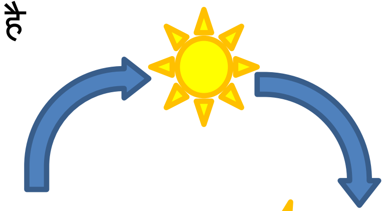

SAME-DAY RESULTS
उसी दिन परिणाम बतायेगा

INGREDIENTS ARE ALREADY COMBINED
सामग्री पहले से ही संयुक्त है

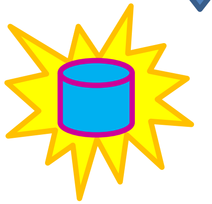

COST PER TEST
प्रति परीक्षण का दाम

₹50

2

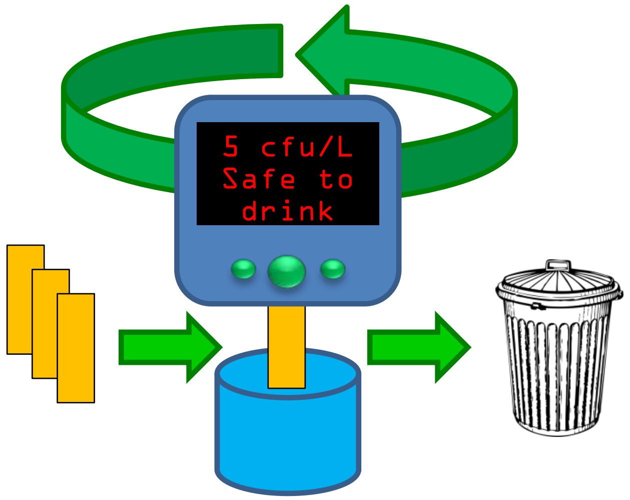

MOSTLY REUSABLE
ज्यादातर पुन: प्रयोज्य

TELLS AMOUNT OF CONTAMINANT
AND RECOMMENDED ACTION
दूषित पदार्थों की मात्रा और कार्य योजना बताता है

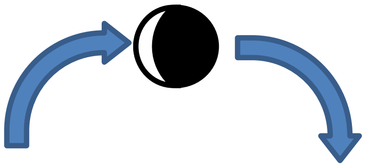

NEXT-DAY RESULTS
अगले दिन परिणाम बतायेगा

INGREDIENTS ARE ALREADY COMBINED
सामग्री पहले से ही संयुक्त है

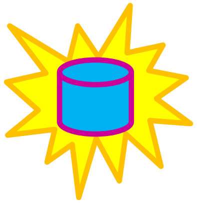

REUSABLE PART COST
पुन: प्रयोज्य भाग का मूल्य

₹500

COST PER TEST
प्रति परीक्षण का दाम

₹25

1

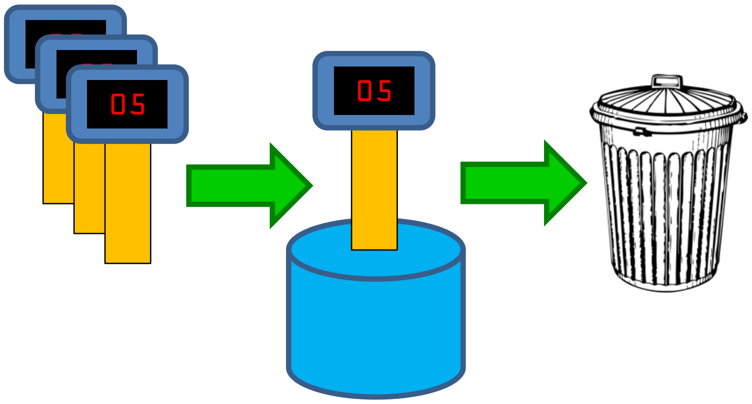

DISPOSABLE
डिस्पोजेबल

TELLS AMOUNT OF CONTAMINANT
दूषित पदार्थों की मात्रा बताता है

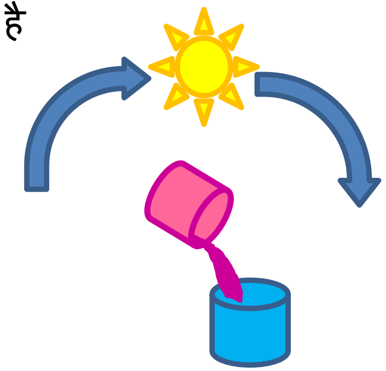

SAME-DAY RESULTS
उसी दिन परिणाम बतायेगा

ADD LIQUID INGREDIENTS
तरल सामग्री जोड़ें

COST PER TEST
प्रति परीक्षण का दाम

₹50

2

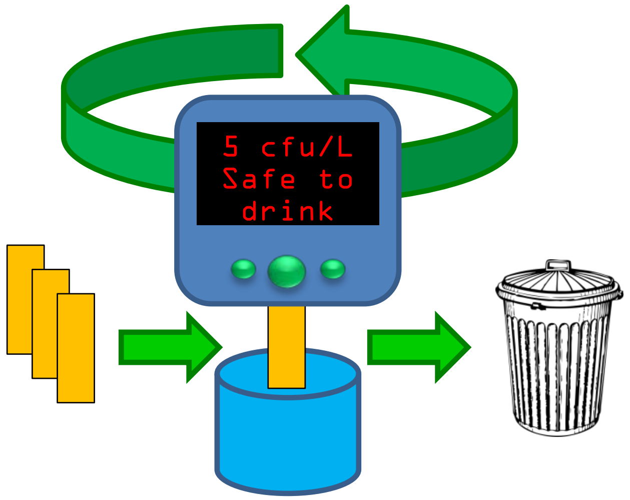

MOSTLY REUSABLE
ज्यादातर पुन: प्रयोज्य

TELLS AMOUNT OF CONTAMINANT AND RECOMMENDED ACTION
दूषित पदार्थों की मात्रा और कार्य योजना बताता है

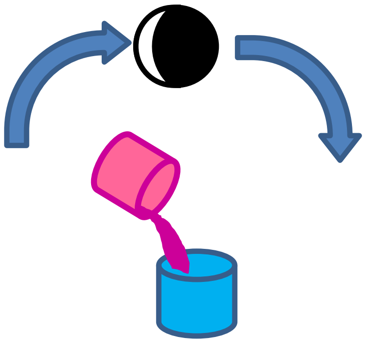

NEXT-DAY RESULTS
अगले दिन परिणाम बतायेगा

ADD LIQUID INGREDIENTS
तरल सामग्री जोड़ें

REUSABLE PART COST
पुन: प्रयोज्य भाग का मूल्य

₹500

COST PER TEST
प्रति परीक्षण का दाम

₹25

1

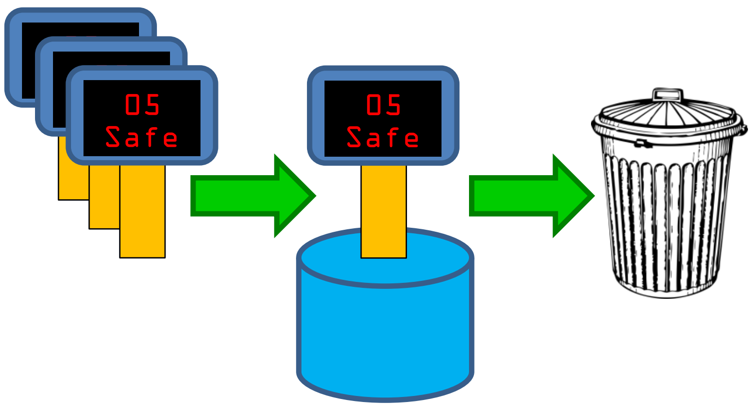

DISPOSABLE
डिस्पोजेबल

TELLS AMOUNT OF CONTAMINANT AND RECOMMENDED ACTION
दूषित पदार्थों की मात्रा और कार्य योजना बताता है

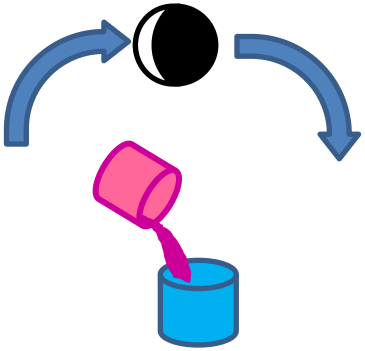

NEXT-DAY RESULTS
अगले दिन परिणाम बतायेगा

ADD LIQUID INGREDIENTS
तरल सामग्री जोड़ें

COST PER TEST
प्रति परीक्षण का दाम ₹100

2

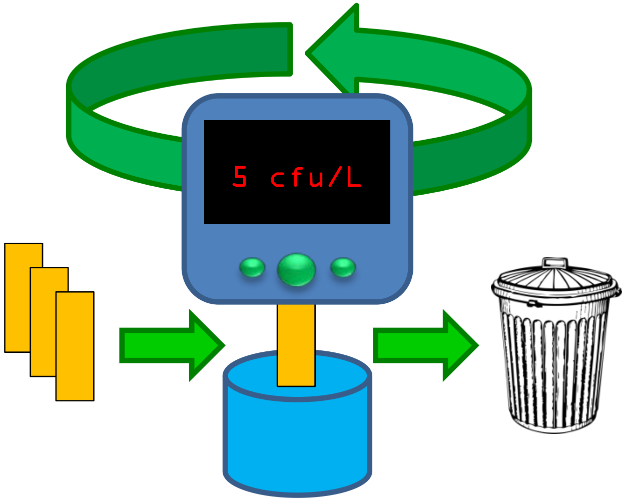

MOSTLY REUSABLE
ज्यादातर पुन: प्रयोज्य

TELLS AMOUNT OF CONTAMINANT
दूषित पदार्थों की मात्रा बताता है

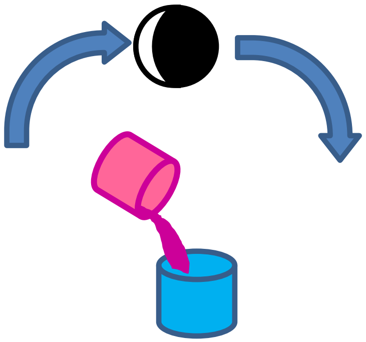

NEXT-DAY RESULTS
अगले दिन परिणाम बतायेगा

ADD LIQUID INGREDIENTS
तरल सामग्री जोड़ें

REUSABLE PART COST
पुन: प्रयोज्य भाग का मूल्य ₹500

COST PER TEST
प्रति परीक्षण का दाम ₹25

1

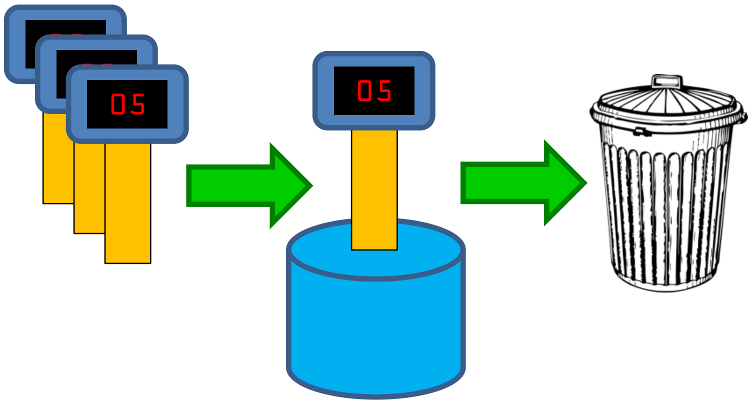

DISPOSABLE
डिस्पोजेबल

TELLS AMOUNT OF CONTAMINANT
दूषित पदार्थों की मात्रा बताता है

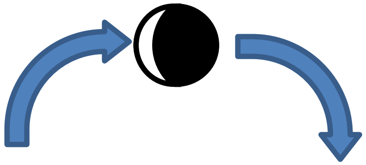

NEXT-DAY RESULTS
अगले दिन परिणाम बतायेगा

INGREDIENTS ARE ALREADY COMBINED
सामग्री पहले से ही संयुक्त है

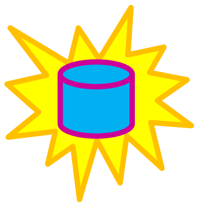

COST PER TEST
प्रति परीक्षण का दाम

₹50

2

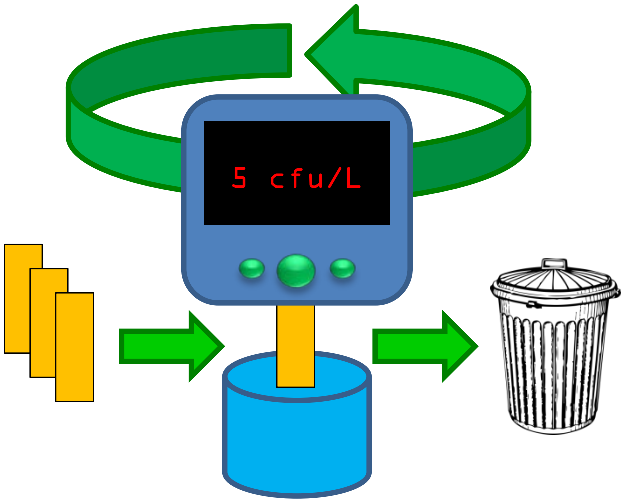

MOSTLY REUSABLE
ज्यादातर पुन: प्रयोज्य

TELLS AMOUNT OF CONTAMINANT
दूषित पदार्थों की मात्रा बताता है

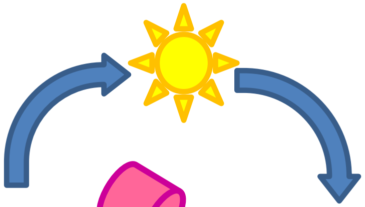

SAME-DAY RESULTS
उसी दिन परिणाम बतायेगा

ADD LIQUID INGREDIENTS
तरल सामग्री जोड़ें

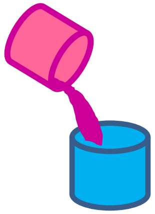

REUSABLE PART COST
पुन: प्रयोज्य भाग का मूल्य

₹500

COST PER TEST
प्रति परीक्षण का दाम

₹25

1

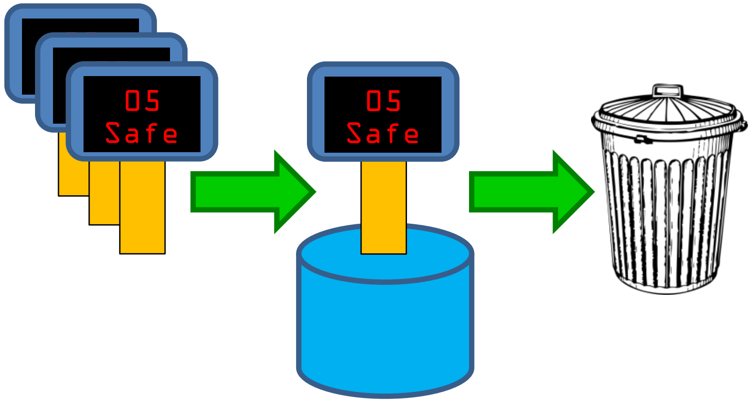

DISPOSABLE
डिस्पोजेबल

TELLS AMOUNT OF CONTAMINANT AND RECOMMENDED ACTION
दूषित पदार्थों की मात्रा और कार्य योजना बताता है

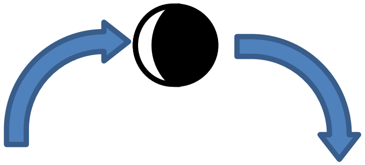

NEXT-DAY RESULTS
अगले दिन परिणाम बतायेगा

INGREDIENTS ARE ALREADY COMBINED
सामग्री पहले से ही संयुक्त है

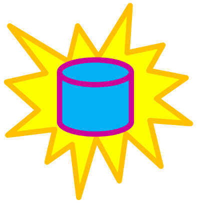

COST PER TEST
प्रति परीक्षण का दाम

₹100

2

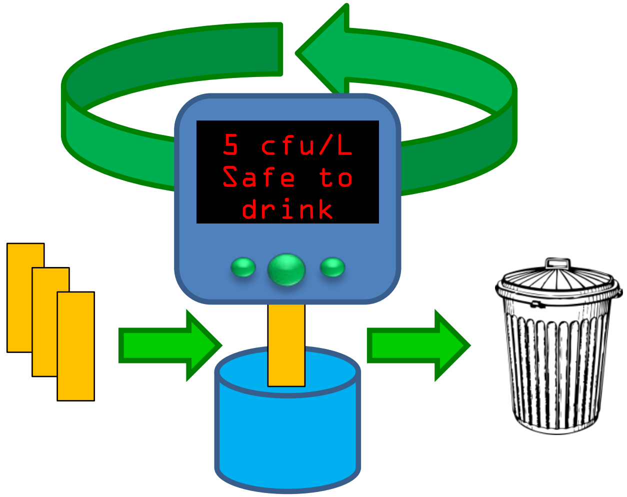

MOSTLY REUSABLE
ज्यादातर पुन: प्रयोज्य

TELLS AMOUNT OF CONTAMINANT AND RECOMMENDED ACTION
दूषित पदार्थों की मात्रा और कार्य योजना बताता है

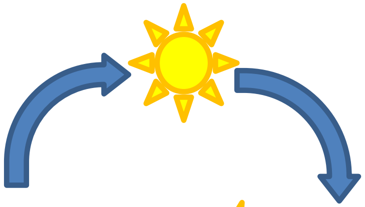

SAME-DAY RESULTS
उसी दिन परिणाम बतायेगा

INGREDIENTS ARE ALREADY COMBINED
सामग्री पहले से ही संयुक्त है

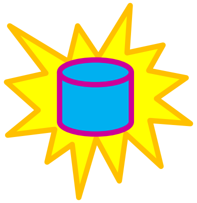

REUSABLE PART COST
पुन: प्रयोज्य भाग का मूल्य

₹500

COST PER TEST
प्रति परीक्षण का दाम

₹25

1

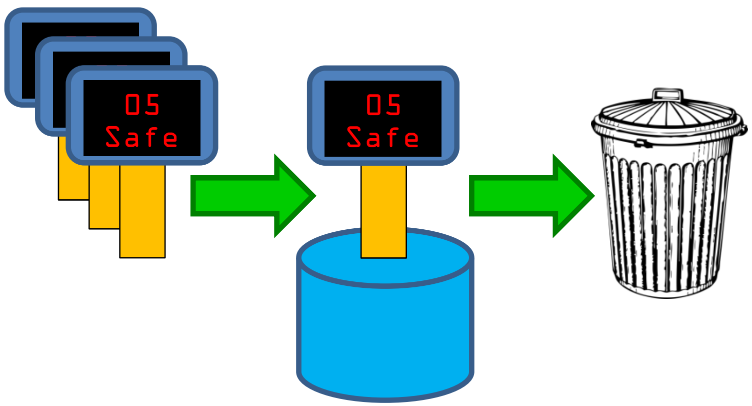

DISPOSABLE
डिस्पोजेबल

TELLS AMOUNT OF CONTAMINANT AND RECOMMENDED ACTION
दूषित पदार्थों की मात्रा और कार्य योजना बताता है

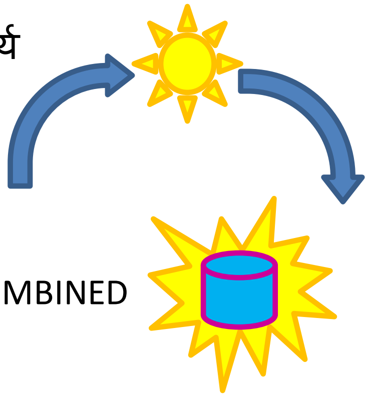

SAME-DAY RESULTS
उसी दिन परिणाम बतायेगा

INGREDIENTS ARE ALREADY COMBINED
सामग्री पहले से ही संयुक्त है

COST PER TEST
प्रति परीक्षण का दाम

₹100

2

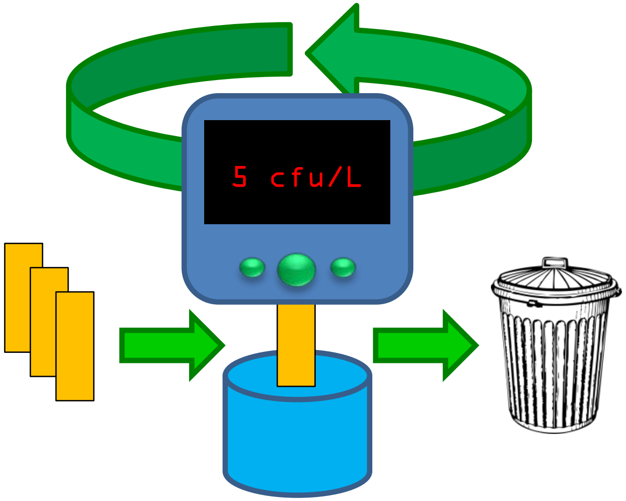

MOSTLY REUSABLE
ज्यादातर पुन: प्रयोज्य

TELLS AMOUNT OF CONTAMINANT
दूषित पदार्थों की मात्रा बताता है

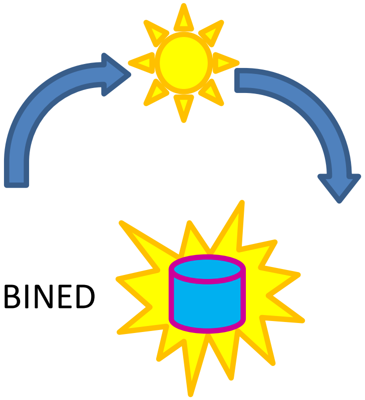

SAME-DAY RESULTS
उसी दिन परिणाम बतायेगा

INGREDIENTS ARE ALREADY COMBINED
सामग्री पहले से ही संयुक्त है

REUSABLE PART COST
पुन: प्रयोज्य भाग का मूल्य

₹500

COST PER TEST
प्रति परीक्षण का दाम

₹25

1

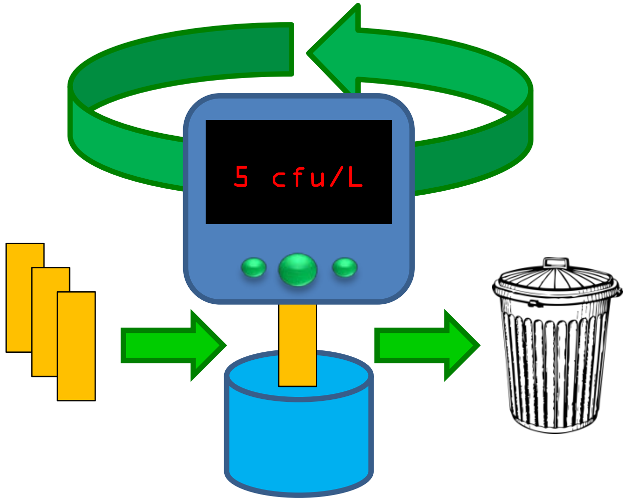

MOSTLY REUSABLE
ज्यादातर पुन: प्रयोज्य

TELLS AMOUNT OF CONTAMINANT
दूषित पदार्थों की मात्रा बताता है

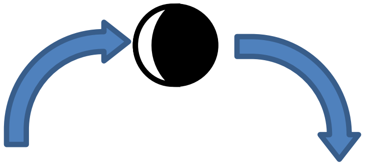

NEXT-DAY RESULTS
अगले दिन परिणाम बतायेगा

INGREDIENTS ARE ALREADY COMBINED
सामग्री पहले से ही संयुक्त है

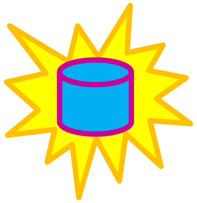

REUSABLE PART COST
पुन: प्रयोज्य भाग का मूल्य ₹1000

COST PER TEST
प्रति परीक्षण का दाम ₹50

2

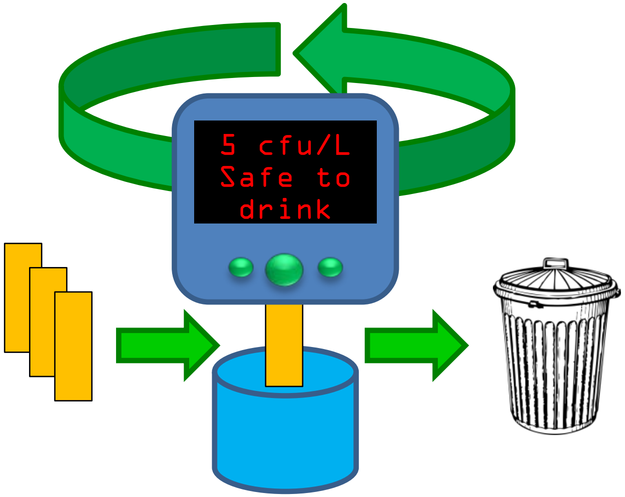

MOSTLY REUSABLE
ज्यादातर पुन: प्रयोज्य

TELLS AMOUNT OF CONTAMINANT AND RECOMMENDED ACTION
दूषित पदार्थों की मात्रा और कार्य योजना बताता है

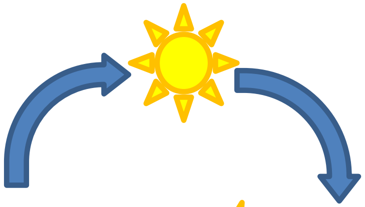

SAME-DAY RESULTS
उसी दिन परिणाम बतायेगा

INGREDIENTS ARE ALREADY COMBINED
सामग्री पहले से ही संयुक्त है

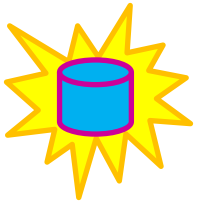

REUSABLE PART COST
पुन: प्रयोज्य भाग का मूल्य ₹500

COST PER TEST
प्रति परीक्षण का दाम ₹25

1

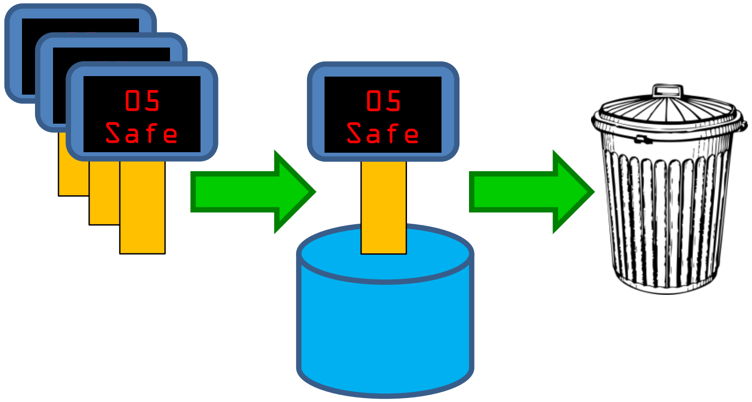

DISPOSABLE
डिस्पोजेबल

TELLS AMOUNT OF CONTAMINANT AND RECOMMENDED ACTION
दूषित पदार्थों की मात्रा और कार्य योजना बताता है

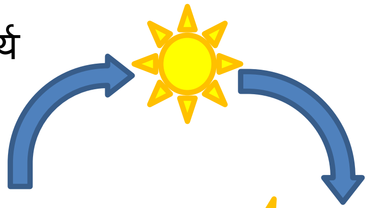

SAME-DAY RESULTS
उसी दिन परिणाम बतायेगा

INGREDIENTS ARE ALREADY COMBINED
सामग्री पहले से ही संयुक्त है

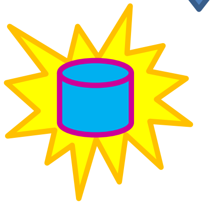

COST PER TEST
प्रति परीक्षण का दाम

₹100

2

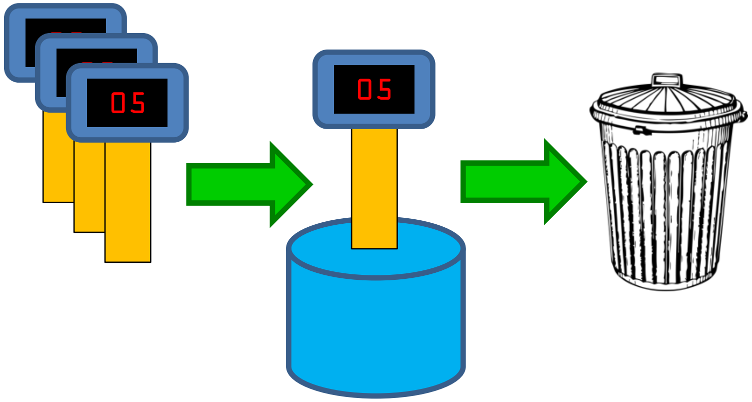

DISPOSABLE
डिस्पोजेबल

TELLS AMOUNT OF CONTAMINANT
दूषित पदार्थों की मात्रा बताता है

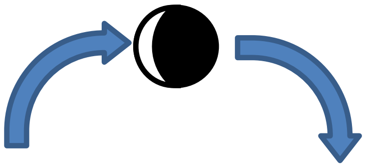

NEXT-DAY RESULTS
अगले दिन परिणाम बतायेगा

INGREDIENTS ARE ALREADY COMBINED
सामग्री पहले से ही संयुक्त है

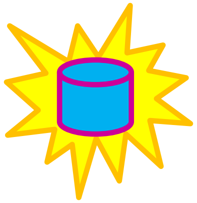

COST PER TEST
प्रति परीक्षण का दाम

₹50

1

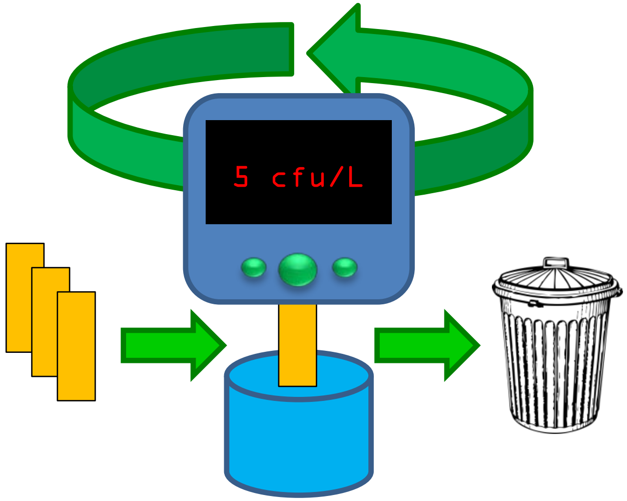

MOSTLY REUSABLE
ज्यादातर पुन: प्रयोज्य

TELLS AMOUNT OF CONTAMINANT
दूषित पदार्थों की मात्रा बताता है

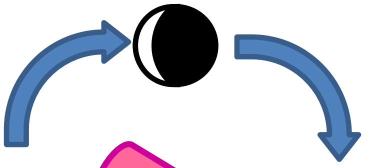

NEXT-DAY RESULTS
अगले दिन परिणाम बतायेगा

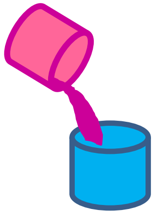

ADD LIQUID INGREDIENTS
तरल सामग्री जोड़ें

REUSABLE PART COST
पुन: प्रयोज्य भाग का मूल्य ₹500

COST PER TEST
प्रति परीक्षण का दाम ₹25

2

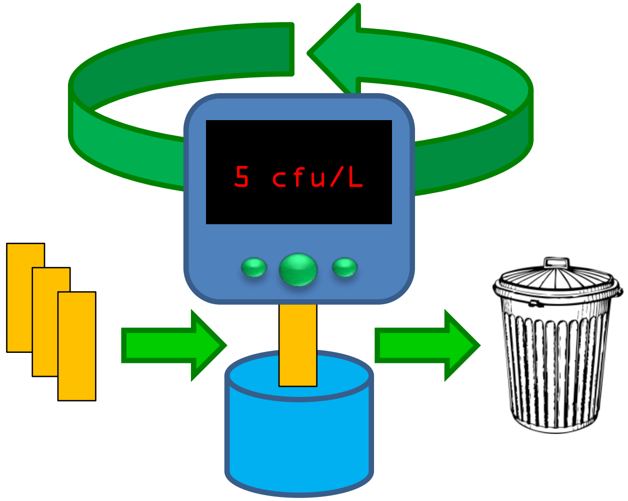

MOSTLY REUSABLE
ज्यादातर पुन: प्रयोज्य

TELLS AMOUNT OF CONTAMINANT
दूषित पदार्थों की मात्रा बताता है

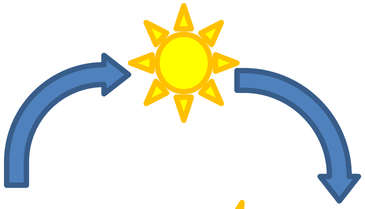

SAME-DAY RESULTS
उसी दिन परिणाम बतायेगा

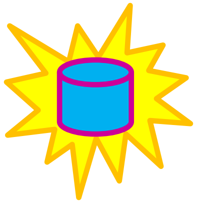

INGREDIENTS ARE ALREADY COMBINED
सामग्री पहले से ही संयुक्त है

REUSABLE PART COST
पुन: प्रयोज्य भाग का मूल्य ₹1000

COST PER TEST
प्रति परीक्षण का दाम ₹50

1

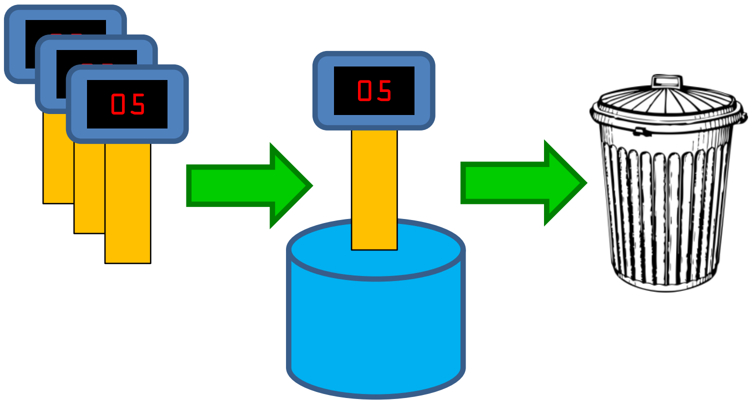

DISPOSABLE
डिस्पोजेबल

TELLS AMOUNT OF CONTAMINANT
दूषित पदार्थों की मात्रा बताता है

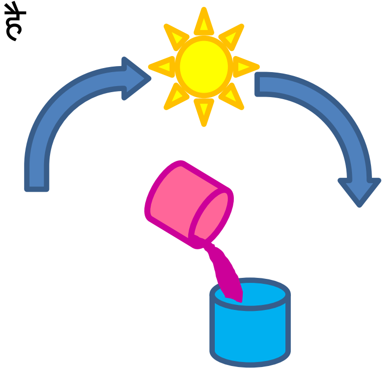

SAME-DAY RESULTS
उसी दिन परिणाम बतायेगा

ADD LIQUID INGREDIENTS
तरल सामग्री जोड़ें

COST PER TEST
प्रति परीक्षण का दाम

₹100

2

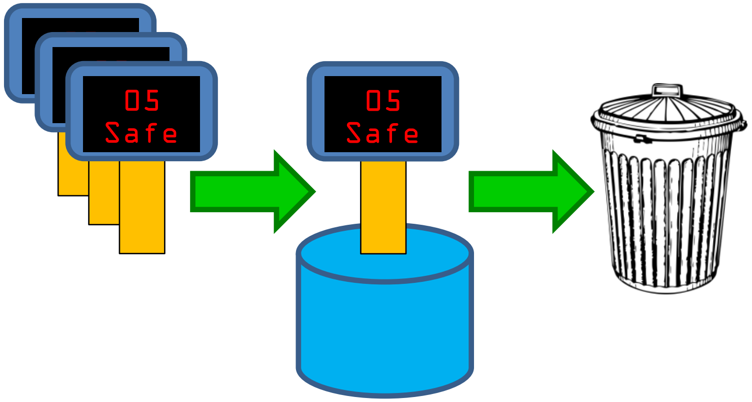

DISPOSABLE
डिस्पोजेबल

TELLS AMOUNT OF CONTAMINANT
AND RECOMMENDED ACTION
दूषित पदार्थों की मात्रा और कार्य
योजना बताता है

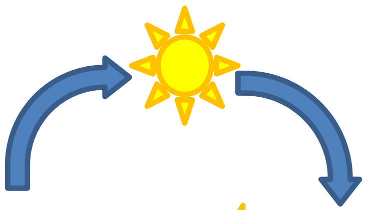

SAME-DAY RESULTS
उसी दिन परिणाम बतायेगा

INGREDIENTS ARE ALREADY COMBINED
सामग्री पहले से ही संयुक्त है

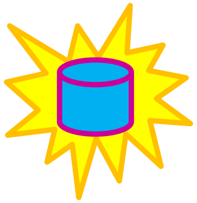

COST PER TEST
प्रति परीक्षण का दाम

₹50

1

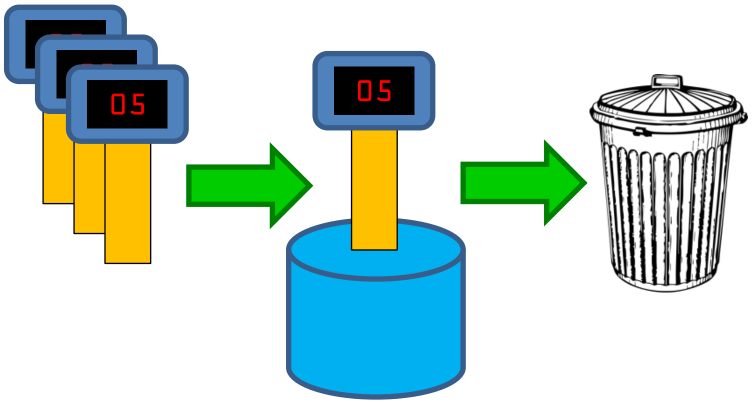

DISPOSABLE
डिस्पोजेबल

TELLS AMOUNT OF CONTAMINANT
दूषित पदार्थों की मात्रा बताता है

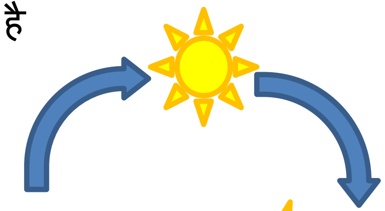

SAME-DAY RESULTS
उसी दिन परिणाम बतायेगा

INGREDIENTS ARE ALREADY COMBINED
सामग्री पहले से ही संयुक्त है

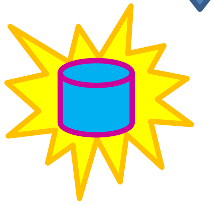

COST PER TEST
प्रति परीक्षण का दाम ₹100

2

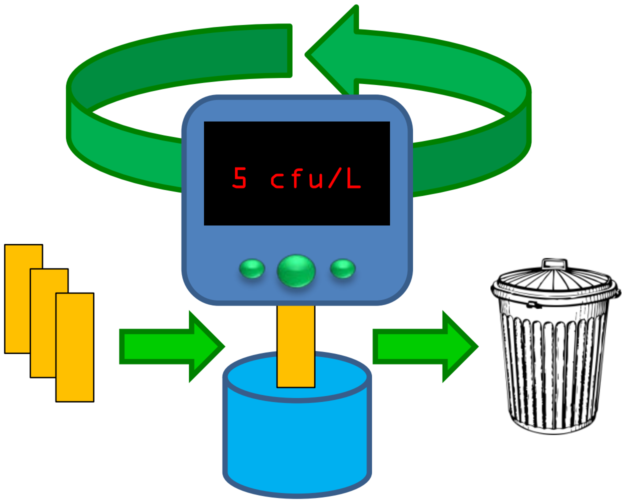

MOSTLY REUSABLE
ज्यादातर पुन: प्रयोज्य

TELLS AMOUNT OF CONTAMINANT
दूषित पदार्थों की मात्रा बताता है

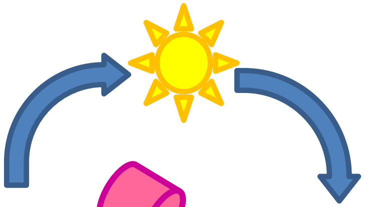

SAME-DAY RESULTS
उसी दिन परिणाम बतायेगा

ADD LIQUID INGREDIENTS
तरल सामग्री जोड़ें

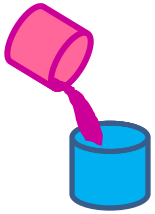

REUSABLE PART COST
पुन: प्रयोज्य भाग का मूल्य ₹500

COST PER TEST
प्रति परीक्षण का दाम ₹25
